# K5 Capsule and Lipopolysaccharide Are Important in Resistance to T4 Phage Attack in Probiotic *E. coli* Strain Nissle 1917

**DOI:** 10.3389/fmicb.2019.02783

**Published:** 2019-11-29

**Authors:** Manonmani Soundararajan, Rudolf von Bünau, Tobias A. Oelschlaeger

**Affiliations:** ^1^Institute for Molecular Infection Biology, Julius Maximilian University of Würzburg, Würzburg, Germany; ^2^Pharma-Zentrale GmbH, Herdecke, Germany

**Keywords:** *E. coli* Nissle 1917, phage resistance, T4 phages, lipopolysaccharide, K5 capsule, probiotics, gastrointestinal infections, Mutaflor

## Abstract

Rapidly growing antibiotic resistance among gastrointestinal pathogens, and the ability of antibiotics to induce the virulence of these pathogens makes it increasingly difficult to rely on antibiotics to treat gastrointestinal infections. The probiotic *Escherichia coli* strain Nissle 1917 (EcN) is the active component of the pharmaceutical preparation Mutaflor^®^ and has been successfully used in the treatment of gastrointestinal disorders. Gut bacteriophages are dominant players in maintaining the microbial homeostasis in the gut, however, their interaction with incoming probiotic bacteria remains to be at conception. The presence of bacteriophages in the gut makes it inevitable for any probiotic bacteria to be phage resistant, in order to survive and successfully colonize the gut. This study addresses the phage resistance of EcN, specifically against lytic T4 phage infection. From various experiments we could show that (i) EcN is resistant toward T4 phage infection, (ii) EcN’s K5 polysaccharide capsule plays a crucial role in T4 phage resistance and (iii) EcN’s lipopolysaccharide (LPS) inactivates T4 phages and notably, treatment with the antibiotic polymyxin B which neutralizes the LPS destroyed the phage inactivation ability of isolated LPS from EcN. Combination of these identified properties in EcN was not found in other tested commensal *E. coli* strains. Our results further indicated that *N*-acetylglucosamine at the distal end of O6 antigen in EcN’s LPS could be the interacting partner with T4 phages. From our findings, we have reported for the first time, the role of EcN’s K5 capsule and LPS in its defense against T4 phages. In addition, by inactivating the T4 phages, EcN also protects *E. coli* K-12 strains from phage infection in tri-culture experiments. Our research highlights phage resistance as an additional safety feature of EcN, a clinically successful probiotic *E. coli* strain.

## Introduction

The healthy human gut is not only home to human cells but also to a complex microbial community which includes bacteria, archaea, protozoa, fungi, and viruses (Lloyd-Price et al., 2016; Manrique et al., 2017). Imbalance in the gut microbial population is termed as dysbiosis and is associated with various gastrointestinal disorders and infections (Lepage et al., 2008; Bien et al., 2013; Molloy et al., 2013; Pham and Lawley, 2014). Gastrointestinal infections are the fourth most common healthcare-associated infection reported in the year 2016–2017 in Europe, with colon and rectal regions of the human intestine documented as the third most common cancer-prone regions (OECD and European Union, 2018) and often cancers in these regions are also associated with localized bacterial infections (Panwalker, 1988; Kumar et al., 2017). At the clinical level, these disorders and infections are approached with combination therapies including antibiotics, hemodialysis, fecal microbial transfer, etc. (Malamud and Wilson, 2000; Goldwater and Bettelheim, 2012; Malikowski et al., 2017). However, usage of antibiotics disturbs the commensal microbiota (Carlet, 2012; Becattini et al., 2016; Miryala and Ramaiah, 2018), and the parallel boom in the emergence of antibiotic-resistant gastrointestinal pathogens worsens the scenario imposing a need for alternative strategies. Usage of probiotics to treat infections dates back to the early 19th century when the scientist Elie Metchnikoff claimed that fermented milk with lactic-acid bacteria suppressed the growth of proteolytic bacteria and hence modified the gut microbiota (Vaughan, 2012). Further, the idea of treating infections with beneficial bacteria was strengthened when German Prof. Alfred Nissle isolated a probiotic *Escherichia coli* strain Nissle from the feces of a soldier who was not infected during a shigellosis outbreak in 1917 (Nissle, 1918, 1925). Later on, this strain was termed Nissle, 1917 (EcN). Since then, EcN is one of the highly used and extensively researched probiotic strains (Wassenaar, 2016) and hitherto, EcN is actively employed in the clinical settings for the treatment of various gastrointestinal disorders (Henker et al., 2007; Arribas et al., 2009; Losurdo et al., 2015; Kotlowski, 2016; Scaldaferri et al., 2016; Sonnenborn, 2016). The successful implementation of EcN as a probiotic is attributed to the lack of pathogenic factors and antibiotic resistance (Grozdanov et al., 2004; Toloza et al., 2015). Moreover EcN also possess several fitness factors such as two anti-bacterial microcins responsible for its antagonistic activity against certain other bacteria (Patzer et al., 2003), seven iron uptake systems assisting in efficient colonization of the gut (Grozdanov et al., 2004; Grosse et al., 2006; Valdebenito et al., 2006; Deriu et al., 2013), three fimbrial determinants aiding in biofilm formation and adhesion to epithelial cells, etc. (Lasaro et al., 2009; Kleta et al., 2014), a H1 type flagella responsible for mucin binding and inducing human beta-defensins (Schlee et al., 2007; Troge et al., 2012),a K5 polysaccharide capsule and a semi-rough lipopolysaccharide (LPS) with a unique O6 antigen involved in immunomodulatory effects (Grozdanov et al., 2002; Hafez et al., 2010; Nzakizwanayo et al., 2015). These fitness factors are said to collectively contribute to the probiotic nature of EcN.

Though EcN’s ability to colonize and outcompete other bacteria in the gut is extensively researched (Hancock et al., 2010), its interaction with the major microbial component of the gut i.e., “phages” are not addressed so far. The ratio of bacteria to phages in the gut is reported to be 1:1 (Mirzaei and Maurice, 2017) and under this circumstance, the encounter of phages by the consumed probiotic bacteria is unavoidable. Such an encounter can lead to a different sequence of events based upon the type of phages. Bacteriophages based on their life-cycle are broadly classified as lysogenic and lytic phages. When a probiotic strain is attacked by lytic phages, the bacteria will be lysed upon the successful infection and hence failing to colonize the gut, whereas if it has encountered a lysogenic phage then there is a probable risk of horizontal transfer of genetic elements encoding toxin or antibiotic resistance that contributes to the pathogenicity or virulence of the strain (Herold et al., 2004; Chen and Novick, 2009; Iversen et al., 2015; Colavecchio et al., 2017). To avoid the event of lysis and genome instability, phage resistance becomes an imperative attribute for any probiotic strain. To review this crucial characteristic of EcN, in this study, we focused on the interactions between EcN and the lytic T4 phages.

T4 phages belong to one of the most common families of phages present in the gut: *Myoviridae* (Kim et al., 2011; Manrique et al., 2016; Lusiak-Szelachowska et al., 2017; Mirzaei and Maurice, 2017) and a lot of T4-like phages have been successfully isolated from human feces (Chibani-Chennoufi et al., 2004; Kim et al., 2010; Hoyles et al., 2014) making them an important challenge for EcN’s survival in the human gut. Phage infection cycle is initiated upon adsorption of phages to specific phage receptors at the host cell surface. There is a great diversity reported in *E. coli* phage receptors which include outer membrane proteins, porins, bacterial capsules, smooth and rough LPS, etc. (Lindberg, 1973; Bertozzi Silva et al., 2016). T4 phage adsorption to *E. coli* is a two-step process, the first is a reversible attachment to LPS which is followed by an irreversible attachment to the phage receptor in the outer membrane of the *E. coli* cell (Gonzalez et al., 2018). It is concluded that an LPS with glucose residues at the distal end of the core oligosaccharide is required for phage adsorption to the cells (e.g., *E. coli* B strain), whereas in case of K-12 strains since the glucose residue at the distal end is masked by heptose, the presence of an outer membrane protein C (Omp C) is required for successful T4 phage adsorption (Mutoh et al., 1978; Washizaki et al., 2016). In contrary to the *E. coli* B and K-12 strains, EcN harbors a unique LPS with a single O6 repeating unit and a K5 capsule which forms the outermost protective layer of EcN. Therefore, one could imagine that the T4 phage interplay with EcN cells will be distinctive in comparison to the other *E. coli* strains.

The main objective of this study was to investigate the interaction of EcN with T4 phages, followed by understanding the resistance and/or phage neutralization mechanisms adopted by EcN to combat the T4 phage attack and to uncover the involvement of EcN’s fitness factors in its phage defense. Besides, we are also interested to address its ability to protect other sensitive *E. coli* strains from T4 infection.

## Materials and Methods

### Bacterial Strains and Oligonucleotides

*Escherichia coli* strains used in this study are listed in Table 1. All strains were grown in Luria-Bertani broth (LB) medium (10 g/l tryptone, 5 g/l yeast, 5 g/l NaCl) at 37°C. Unless otherwise mentioned, the source of the bacterial strains used in the study is the strain collection of the Institute for Molecular Infection Biology, Würzburg. The oligonucleotides used in this study are mentioned in Table 2. For the PCR reactions, unless otherwise specified, the 2× PCR MM (Cat no: K0171, Thermo Fischer Scientific) was used along with the respective primer pair. The PCR conditions were adopted according to the manufacturer’s protocol.

**TABLE 1:** *E. coli* strains used in this study.

***E. coli* strain**	**Serotype**	**Description**
*E. coli* Nissle 1917 (EcN)	O6:K5: H1	Non-pathogenic probiotic strain, pMUT1, pMUT2, microcins H47/M, F1C-fimbria, type 1 fimbria, curli Source: Pharma-Zentrale GmbH, Herdecke
SK22D	O6:K5: H1	A *mchCDEF* deletion mutant of EcN (no microcins)
EcN Δ*k5*	O6:K5: H1	A *kps* deletion mutant of EcN which lacks the entire determinant spanning from *kpsF* to *kpsM* (17521 bp)
*E. coli* K-12 MG1655	O^–^:H48: K^–^	K-12 wildtype strain, F^–^, λ^–^, ilvG-, rfb-50, rph-1
*E. coli* K-12 HB101	O^–^:H48: K^–^	K-12 derivative; F^–^, λ^–^, mcrB, mrr, hsdS20 (r_*B*_^–^ m_*B*_^–^), recA13, leuB6, ara-14, proA2, lacY1, galK2, xyl-5, mtl-1, rpsL20 (SmR), glnV44
*E. coli* K-12 DH5α	O^–^:H48: K^–^	K-12 derivative; F^–^, endA1, hsdR17 (rk^–^, mk^–^), supE44, thi-1, recA1, GyrA96, relA1, λ-, Δ(lacZYA-argF)U169, Φ80dlacZ ΔM15, deoR, nupG
*E. coli* SE11	O152: H28	Commensal strain isolated from healthy adult Source: Japan Collection of Microorganisms (JCM 16574) (Oshima et al., 2008)
*E. coli* SE15	O150: H5	Commensal strain isolated from healthy adult Source: Japan Collection of Microorganisms (JCM 16575) (Toh et al., 2010)
*E. coli* CFT073	O6: K2: H1	Uropathogenic *E. coli* strain, human isolate, hemolysin, type IV Pili, S and P fimbrial adhesins, a close relative to EcN

**TABLE 2:** Oligonucleotides used in this study.

**Name**	**Sequence (5′–3′) concentration [nM] – 500**	**Amplicon size [bp]**	**Application**
T4_*ndd*_F	CCTCACTGGCGTCCGAAGAC	2*580	For localization of T4 phage DNA. The primer pair amplifies the T4 phage specific *ndd* gene
T4_*ndd*_R	TCATGCGGCCTTGGAGTAGAA		
pKD3_F	ACACGTCTTGAGCGATTGTG	2*1098	Primers to screen pKD3 plasmid that is used as internal standard in T4 phage DNA localization PCR
pKD3_R	AGCCTCTCAAAGCAATTTTC		
K5_1	AGTGAAGGAAGGCCCGGAAG	1083 bp amplicon in EcN Δ*k5* mutant, no amplicon in case of EcN wildtype	External primers for genotypic verification of the capsule negative mutant. Primer K5_1 bind 694 bp upstream of *kpsF* and K5_2 binds 391 bp upstream of *kpsM* of the capsule (*Kps*) determinant in EcN
K5-2	ATCAATCGCGTGCGTTCTGG		
K5_3	GAACGGTGCGGCAGTCAACG	1045 bp amplicon in EcN wildtype, no amplicon in case of EcN Δ*k5* mutant	Internal primers for genotypic verification of the capsule negative mutant. Primer pair binds inside the Capsule (*Kps*) determinant of EcN
K5_4	GACGATGTCCCCACACGGCG		

### Propagation of T4 Phages

The T4 phage was propagated by adding 100 μl of T4 phage stock (∼10^7^ Pfus/ml) to 20 ml of *E. coli* K-12 MG1655 culture in its early log growing phase (OD_600_∼0.3). The culture was further incubated at 37°C in a rotary shaker (200 rpm) until clear lysis was observed (∼5 to 6 h). Chloroform (2%) was added to the lysate and incubated for 15 min at room temperature (RT) and centrifuged (4696 × *g*, 10 min, 4°C). The phages were then isolated by sterile filtration (0.22 μm PALL filter, Cat no: 514-4131, VWR) of the supernatant. For microscopy experiments, T4 phage lysate was further concentrated as described in Bonilla et al. (2016). Briefly, phage lysate was concentrated by repeated centrifugation in Amicon^®^ Ultra-15 centrifugal filter units, Ultracel 100 KDa membrane (Cat no: UFC910008, Millipore). By this technique, we were able to produce phage stocks of high concentration (up to 10^15^ Pfus/ml).

### Bacteria and Phage Coincubation Conditions

T4 phage lysate was incubated with bacterial strains as described in Bury et al. (2018). In short, the OD_600_ of bacterial overnight cultures (ONC) was determined and the cells were collected by centrifugation at 4696 × *g* for 10 min at RT. The bacterial pellet was resuspended in fresh LB medium to obtain ∼10^9^ Cfus/ml. 100 μl of a phage extract (∼10^9^ Pfus/ml) were used to set up mono; co; or tri-cultures with 100 μl of EcN and/or *E. coli* K-12 strains in a 24 well plate. Each well was adjusted to a final volume of 1 ml with LB medium. The plates were incubated in a static manner for the desired amount of time at 37°C. The Cfus/ml were determined by plating serial dilutions in 0.9% saline on 1.5% LB agar plates. The samples were sterile filtered (0.22 μm PALL filter, Cat No: 514-4131, VWR) and serially diluted in 0.9% saline for further analysis.

### Phage Titer Determination by Phage Plaque Assay

Before and after the coincubation studies, phage titers were determined by phage plaque assay (PPA). For this, 200 μl of an MG1655 ONC (OD_600_ 3.0–4.0) was added to 0.7% LB agar (hand bearable warmth), mixed well and poured on top of a 1.5% LB agar plate. The phage particles in the sterile filtrate were determined as plaque-forming units/ml (Pfus/ml) by dropping 10 μl of phage sample dilutions on the MG1655 indicator plate.

### Confocal Microscopic Examination of T4 Phage and *E. coli* Incubation

For the confocal microscopy, the bacteria-phage infection method was adapted from Zeng and Golding (2011) and modified for T4 phages as described here: T4 phages (∼10^11^ Pfus/ml) were stained with 0.5 μg/ml DAPI and incubated for 10 min at RT in dark. The excess DAPI was removed by dialyzing the phages using Slide-A-Lyzer mini dialysis cassette (2 KDa MWCO, Cat no: 69580, Thermo Fisher Scientific) against 100-fold LB medium in a 50 ml falcon tube (4 h, 4°C, rolling). Meanwhile, the LB agarose slabs were prepared as described in Zeng and Golding (2011). After the dialysis, 100 μl of DAPI stained T4 phages were mixed with 500 μl of mid-log growth phase *E. coli* cells (OD_600_∼0.5) and incubated for 30 min on ice to facilitate the binding. Subsequently, the infection was carried out at 37°C for 5 min. The phage-bacteria mixture was immediately pelleted down (8000 rpm, 5 min) in a tabletop microcentrifuge and the pellets were resuspended in 50 μl LB medium and 2 μl was placed on the agarose slab and it was covered with a coverslip (24 × 50 mm) after a minute. The slide was carefully mounted on the microscope stage and imaging was done at a high-magnification (100× objective) in Leica microscope-TCS MP5 (Leica Microsystems, Germany).

### Transmission Electron Microscopic Examination of *E. coli* Incubated With T4 Phages

For negative staining, T4 phages were incubated with *E. coli* at an MOI (multiplicity of infection) of 100:1 for 1 h at 37°C and the samples were then fixed with 0.5% glutaraldehyde and stained with 0.5% uranyl acetate on a copper grid. The image was captured in JEOL JEM-2100 TEM at Zentrale Abteilung für Mikroskopie, Biocenter, Julius-Maximilians Universität Würzburg.

### Localization of T4 Phage DNA by T4 Specific PCR

To determine if the T4 phages are bound to EcN cells or localized in the supernatant, the *ndd* gene-specific PCR was performed with T4_*ndd* _F and T4_*ndd*_R primer pair. For this, 1 ml of *E. coli* cells incubated with T4 phages at a MOI of 1:1 for 24 h, static at 37°C were centrifuged (4696 × *g*, 10 min, RT). The pellets were washed twice with 0.9% saline and were then resuspended in 1 ml of fresh LB medium. Similarly, the supernatant from coincubation was sterile filtered. As an internal control, 1 μl of plasmid pKD3 was used along with primer pair pKD3_F and pKD3_R (amplicon size: 1098 bp) in each PCR reaction and 2 μl of EcN pellet and supernatant samples were used as a template for PCR with 2× PCR Master Mix (Cat no: K0171, Thermo Fischer Scientific) and PCR condition was adopted according to the manufacturer’s protocol as described in the Table 3. The amplicons were tested on 2% agarose gel.

**TABLE 3:** PCR conditions for localization of T4 phage DNA by T4 specific PCR.

**PCR reaction mix**	**1×**	**PCR condition**
2× PCR MM	12.5 μl	Step 1: 95°C for 5 min
T4_*ndd*_F	1.25 μl	Step 2: 95°C for 30 s
T4_*ndd*_R	1.25 μl	Step 3: 55°C for 1 min
Plasmid pKD3	1 μl (30 ng)	Step 4: 72°C for 2 min
pKD3_F	1.25 μl	Step 2 to 4–35 cycles
pKD3_F	1.25 μl	Step 5: 72°C for 10 min
Template	2 μl	Step 6: hold at 4°C
Water	Upto 25 μl	

### Processing of *E. coli* Culture and Supernatant for Further Biochemical Studies

To understand the mechanism(s) of T4 phage inactivation by EcN, *E. coli* strains were subjected to different treatments as mentioned below. (i) Heat-killing: 10 ml of 24 h static cultures were pelleted at 4696 × *g* for 10 min at RT and washed twice with 500 μl of 0.9% saline. The pellets were resuspended in LB medium to obtain 10^10^ Cfus/ml and thereon heat-killed for 1 h at 100°C. The supernatants of heat-killed cells were sterile filtered (0.22 μm PALL filter, Cat no: 514-4131, VWR). (ii) Concentrated supernatant: supernatants of 24 h *E. coli* static cultures were sterile filtered and concentrated approximately 10 times (10×) with Vivaspin Turbo 15 (MWCO: 5 KDa, Cat no: VS15T11, Sartorius). (iii) LPS destruction: Further to understand the involvement of LPS, *E. coli* heat-killed cultures (10^10^ Cfus/ml) or supernatants were incubated with 25 μg/ml polymyxin B (PMB) for 1 h at 37°C. 100 μl of the differentially treated *E. coli* strains or supernatants were added to 100 μl of phages, to a final volume of 1 ml with LB medium and incubated for 24 h at 37°C in a 24 well system. In the case of PMB treatment, 25 μg/ml PMB was used also in the coincubation.

### Phenotypic Verification for the Lack of K5 Polysaccharide Capsule in EcN Δ*k5* Mutant

To confirm the absence of K5 capsule in EcN Δ*k5* mutant, PPA was performed with K5 capsule specific phages (Cat No:60759, Statens Serum Institut). For this, 200 μl of *E. coli* cultures were mixed with 0.7% LB agar and poured on a 1.5% LB agar plates. After solidification, 10 μl of serially diluted K5 capsule specific phage lysate was spotted on the lawn and the plates were carefully incubated for 24 h at 37°C.

### LPS Isolation

To investigate the role of LPS in T4 phage inactivation, LPS was isolated from *E. coli* strains by hot-phenol extraction method as described in Rezania et al. (2011). The isolated LPS was further quantified in a 12% TruPAGE^TM^ Precast Gels (Cat no: PCG2010-10EA, Sigma-Aldrich) followed by an LPS specific staining with Pro-Q^®^ Emerald 300 Lipopolysaccharide Gel Stain Kit (Cat no: P20495, Thermo Fisher Scientific) according to the manufacturer’s instruction.

### T4 Phage Adsorption Study

In order to decipher the T4 phage adsorption to *E. coli* cells at a molecular level, T4 phages were added to mid-log phase (OD_600_∼0.5) EcN and MG1655 culture at an MOI of 0.01 (100 times more *E. coli* than phages) in the presence and absence of 0.6 M *N*-acetylglucosamine (GlcNAc). Pfus/ml were determined by PPA from the sterile filtrate of the culture that was taken at different time points (0, 1, 3, 6, 9, 12 and 30 min) after the T4 phage addition.

### Statistical Analysis

The experiments were performed independently for at least 3 times in triplicates. Data are represented as mean ± SD. For the statistical analysis of the experimental data, we used GraphPad Prism^®^ version 8. The unpaired *t*-test was used for the significance test. For Figures 6, 8A, 9 the significance was calculated by multiple *t*-tests.

## Results

### EcN Is Resistant to Lytic T4 Phage Infection

**FIGURE 1:**
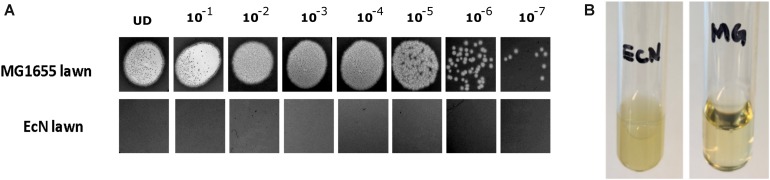
T4 phages sensitivity test for *E. coli* strains MG1655 and EcN by **(A)** phage plaque assay: 10 μl of serially diluted [undiluted (UD) to 10^– 7^ Pfus/ml] T4 phage lysate was spotted on either MG1655 or EcN lawn in 0.7% LB agar and incubated at 37°C for 24 h, static. **(B)** Liquid culture assay: 100 μl of 10^9^ Pfus/ml T4 phage lysate was added to mid-log growing phase EcN or MG1655 culture (OD_600_∼0.5) and incubated at 37°C for 4 h, 180 rpm. Imaging was performed with Canon PowerShot SX260 HS and processed with ImageJ 1.50i.

**FIGURE 2:**
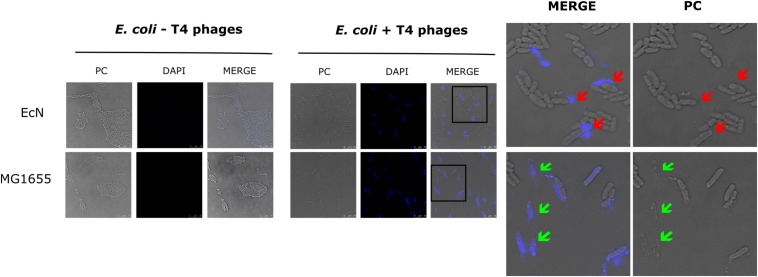
Confocal micrograph showing intact EcN cells after incubation with T4 phages: *E. coli* cells (EcN or MG1655) were mixed with 100 μl of DAPI (0.5 μg/ml) stained T4 lysate or medium at ratio of *E. coli*: T4∼10:1 and incubated on ice for 30 min, followed by incubation at 37°C for 5 min. Imaging was performed at 100× magnification as described in the “Materials and Methods” section. PC: phase contract channel, DAPI: DAPI channel, MERGE: PC + DAPI. *E. coli* -T4 phages (left): EcN or MG1655 cells incubated with medium +0.5 μg/ml DAPI, *E. coli* +T4 phages (middle): EcN or MG1655 cells incubated with T4 phages +0.5 μg/ml DAPI. In zoomed-in version, red arrows point out intact EcN cells, DAPI signal (blue) around the cells **(top row)** and green arrows point out lysed MG1655 cells, DAPI signal (blue) inside the cells **(bottom row)**.

**FIGURE 3:**
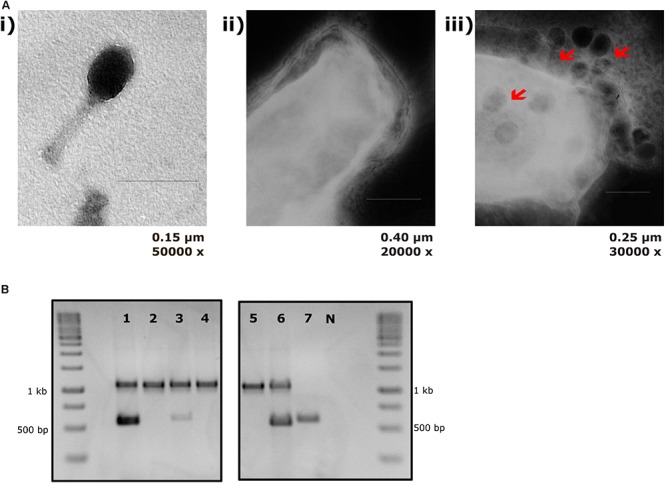
T4 phage attachment to EcN cells: **(A)** Transmission electron micrograph displaying T4 phages attached to EcN cells: T4 phages were incubated with *E. coli* (100:1) at 37°C for 2 h and then fixed with 0.5% glutaraldehyde and stained with 0.5% uranyl acetate. Imaging was performed in TEM at different magnifications. Length of the bar is mentioned under each picture. **(i)** T4 phage incubated with medium **(ii)** EcN incubated with medium **(iii)** EcN incubated with T4 phages and the red arrows point out the T4 phages that are attached to EcN cells. **(B)** Localization of T4 phage DNA in EcN after co-incubation by T4 phage specific PCR: The pellet and the supernatant of EcN incubated with T4 phages for 24 h were used as a template (2 μl/each) in a T4 specific PCR with the primer pair T4_*ndd*_F and T4_*ndd*_R (580 bp). Plasmid pKD3 (1 μl) was used as internal standard along with primer pair pKD3_F and pKD3_R (1098 bp). T4 specific PCR was performed with 2× PCR Master Mix (Cat no: K0171, Thermo Fischer Scientific) and PCR conditions are mentioned in the Table 3. Lane details 1: EcN + T4 phage_pellet, 2: EcN + LB medium_pellet, 3: EcN + T4 phage_supernatant, 4: EcN_supernatant + LB medium, 5: LB medium control, 6: LB medium + T4 phage, 7: T4 phage control (no pKD3), N: negative control, M: GeneRuler 1 kb DNA Ladder (Cat no: SM0311, Thermo Scientific). The agarose gel pictures displayed here are cropped image of same gel. The image was cropped and presented using ImageJ 1.50i for clear understanding.

The sensitivity of EcN for T4 phage infection was tested by standard PPA which revealed the complete T4 phage resistance in EcN. There was neither a lysis zone nor were single plaques detected when serial dilutions of a T4 phage lysate were spotted on a lawn of EcN (Figure 1A). Likewise, when T4 phages were added to mid-log grown cultures of EcN or MG1655, after 4 h of incubation at 37°C, the EcN culture was turbid, indicating the insensitivity to T4 phages (Figure 1B). On the contrary to EcN, in PPAs and liquid culture experiments, MG1655 was clearly lysed upon T4 phage addition. In order to get some insight into the mode of T4 phage resistance in EcN, samples of *E. coli* and T4 phage coincubation cultures were examined by microscopy. For this, mid-log phase grown *E. coli* cultures were added to DAPI stained T4 phages and then incubated for 5 min at 37°C prior to which the coincubation was kept on ice for 30 min. Confocal microscopic examination pointed out a clear DAPI signal (Figure 2) around intact EcN cells. However, for MG1655 incubated with T4 phages, prominent cell lysis was visible and the DAPI signal was localized within the MG1655 cells. These results suggested that T4 phages still bind to EcN without causing lysis. In contrast, MG1655 has undergone lysis in the presence of T4 phages. *E. coli* and T4 phage coincubation samples were negatively stained and examined under a transmission electron microscope to achieve more detailed pictures of T4 interaction with the *E. coli* strains. The EM micrographs (Figure 3A) supported the finding from confocal microscopy. Specifically, the definite attachment of T4 phages to EcN was clearly observed (Figure 3Aiii) thus confirming the fact that T4 phages bind to EcN cells during coincubation, but unlike MG1655, EcN cells are not lysed. Moreover, T4 phage specific PCR suggested that after 24 h of incubation with EcN, the T4 phage DNA was found more frequently associated with EcN cells than in the cell-free supernatant of the coincubation reaffirming the T4 phage binding to EcN (Figure 3B).

### K5 Polysaccharide Capsule Protects EcN From T4 Phage Infection

**FIGURE 4:**
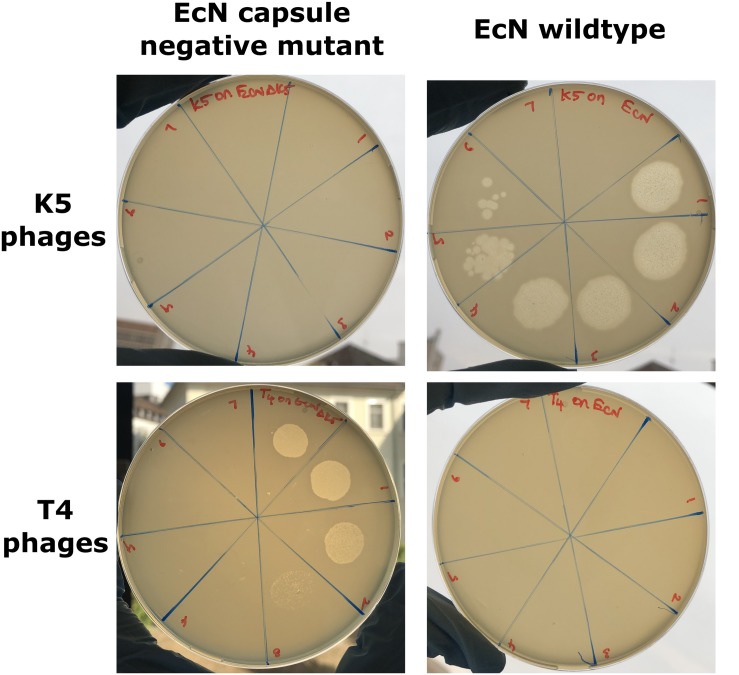
Phage plaque assay plates demonstrating the role of the K5 polysaccharide capsule in EcN’s T4 phage resistance: 10 μl of serially diluted K5 specific phages **(top row)** or T4 phages **(bottom row)** were spotted on the lawn of either EcN Δ*k5* or EcN wildtype in 0.7% LB agar and incubated at 37°C for 24 h, static. Imaging was performed with Canon PowerShot SX260 HS and processed with ImageJ 1.50i.

The K5 polysaccharide capsule, the outermost layer in EcN might either be a binding partner for T4 phages and/or protect EcN against T4 phage infection. To test the role of EcN’s capsule for its resistance to T4 phage infection the EcN Δ*k5* strain was employed in the PPA. This mutant lacks the entire capsule (*kps*) determinant which is approximately 17.9 kb in size. This deletion was confirmed by PCR with K5_1 and K5_2 external primers and K5_3 and K5_4 internal primers (Supplementary Figure S1) and in addition, the lack of capsule in EcN Δ*k5* was verified employing K5 capsule specific phages in PPAs. K5 specific phages could actually infect the *E. coli* strains with K5 capsule producing clear lysis zones in K5 capsule expressing EcN lawns. However, the EcN Δ*k5* strain was completely resistant to these phages as demonstrated by the lack of any lysis zones (Figure 4_top row). Subsequently, the necessity of the K5 polysaccharide capsule with regard to T4 phages infection was investigated. Interestingly, T4 phages spotted efficiently on EcN Δ*k5* lawn in contrast to an EcN wildtype lawn (Figure 4_bottom row) suggesting an important role of the K5 polysaccharide capsule in T4 phage resistance of EcN.

### EcN Cells and Cell-Free Supernatant Inactivate T4 Phages

**FIGURE 5:**
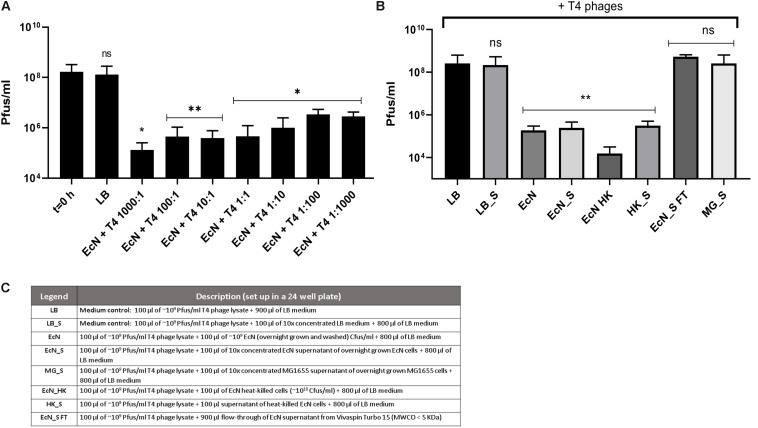
Effect of EcN cells and supernatant on T4 phage titer reduction: EcN cells were incubated with T4 phages for 24 h in static at 37°C at various MOI as mentioned in the graph. After 24 h of incubation, the samples were sterile filtered, and Pfus/ml were determined by phage plaque assay **(A)**. EcN samples were processed as described in the “Materials and Methods” section: 8, and processed samples were coincubated with T4 phages as in panel **(A)** and Pfus/ml were determined by phage plaque assay **(B)**. Legends of the graph B describing the processed *E. coli* samples are presented in the table in panel **(C)**. The asterisks depict the statistical significance of the different samples when compared to the control with LB medium. ns – not significant, ^∗^*p* < 0.05, ^∗∗^*p* < 0.01.

**FIGURE 6:**
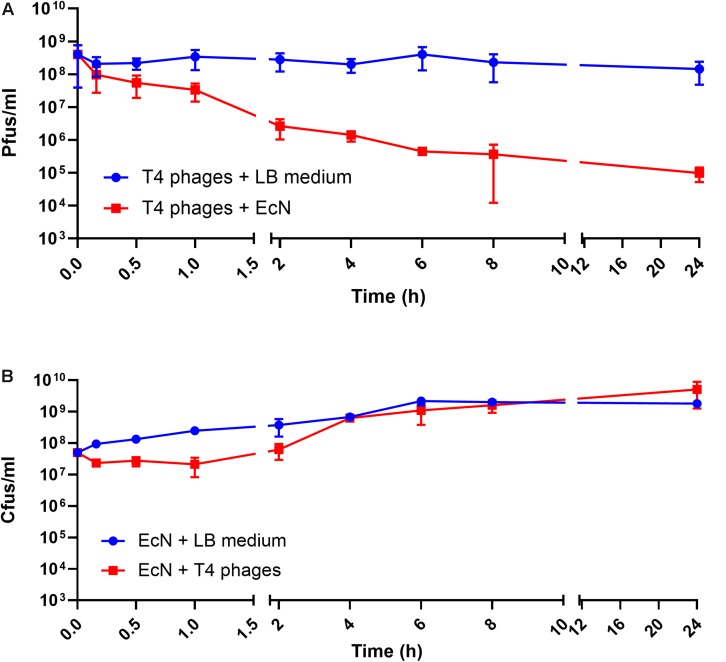
Kinetics of T4 phage inactivation by EcN cells: 100 μl of EcN cells was incubated with 100 μl of T4 phages at an MOI of 1:1 in 1 ml LB medium at 37°C, static. Pfus/ml **(A)** and Cfus/ml **(B)** were determined. The blue line indicates Pfus/ml of T4 phages and Cfus/ml of EcN in LB medium, the red line indicates Pfus/ml of T4 phages incubated with EcN cells and Cfus/ml of EcN incubated with T4 phages.

Recently it has been reported that EcN is able to inactivate *stx*-phages (Bury et al., 2018). We wondered if EcN might also inactivate T4 phages. Therefore, a potential phage inactivation ability of EcN cells was tested by incubating washed, overnight grown EcN cells with T4 phages at various MOI followed by determination of the phage titer. The results (Figure 5A) disclosed an impressive phage titer reduction of up to 1000-fold after 24 h of incubation. Noteworthy, a phage titer reduction of ∼100-fold was visible even when there were 1000 times more phages than EcN incubated together. From the results of coincubation studies with processed EcN cells (EcN) and cell-free supernatant (EcN_S) (Figures 5B,C), it became clear that heat-killed EcN cells (EcN HK) could reduce the T4 phage titer by even ∼10-fold more compared to live EcN cells. Furthermore, the EcN_S which was sterile filtered and 10× concentrated (Vivaspin, MWCO: 5 KDa), as well as supernatant of heat-killed EcN (HK_S), was able to reduce the phage titer as efficiently as live EcN cells. In contrast, the concentrated supernatant from MG1655 (MG_S) did not affect the T4 phage titer, emphasizing that the factors mediating the inactivation of T4 phages could be specific for EcN supernatant. In addition, flow-through of EcN supernatant from Vivaspin (EcN_S FT) did not influence the T4 phage titer on coincubation which excludes the involvement of low molecular weight (less than 5 KDa) components of EcN supernatant in T4 phage inactivation. To apprehend the mechanism of T4 phage inactivation by EcN cells, as a first step, phage titer reduction by EcN was determined after various time points of coincubation (Figure 6A). The results revealed a rapid inactivation of T4 phages by EcN starting with ∼6.3-fold reduction in Pfus/ml already after 30 min of coincubation which gradually increased to 103-fold reduction after 2 h and up to 1400-fold after 24 h of incubation. Correspondingly, the Cfus/ml of EcN in the presence and absence of T4 phages were also determined at each time point (Figure 6B). The graph indicates that there is a lag in EcN’s growth in early time points of coincubation until 1 h, however, after 2 h of coincubation, a shoot up in EcN’s Cfus/ml was observed and there was no significant difference of Cfus/ml between EcN cultures in the presence or absence of T4 phages already after 4 h of incubation. However, the reasons for the growth retardation of EcN in the presence of phages can be hypothesized based on the results from transcriptomic analysis of EcN incubated with T4 phages for 2 h which is presented in the Supplementary Data Sheet S1 (Supplementary Figures S2i,ii and Supplementary Tables S2, S3). Heavy downregulation of genes corresponding to major metabolic pathways was observed and this might be one reason for the growth lag in the early hours of coincubation. This downregulation and growth retardation observed in EcN upon the incubation with T4 phages is consistent with the previously reported inhibition in the cellular mechanisms of host cell by T4 ghost bacteriophages (Winkler and Duckworth, 1971; Fukuma and Kaji, 1972; Vallee et al., 1972). These studies have shown that despite the absence of DNA injection, T4 ghost cells disrupted the host cellular activities which corresponds to our observation.

### LPS Mediated Inactivation of T4 Phages by EcN

**FIGURE 7:**
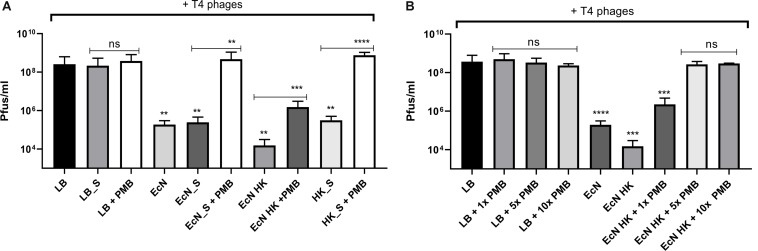
T4 phage inactivation by differentially treated EcN samples: Graph **(A,B)** illustrate the results of coincubation studies performed under various conditions as described: processed EcN samples were further treated with 25 μg/ml polymyxin B (PMB) for 1 h at 37°C and used for the coincubation studies. Pfus/ml were determined after 24 h at 37°C using standard phage plaque assay **(A)**. Legends of the graph are described in Figure 5C. The heat-killed EcN cells were further treated with increasing concentration of PMB: 1× – 25 μg/ml, 5× – 125 μg/ml, 10× – 250 μg/ml. As a control, LB medium was treated with the same concentrations of PMB and used in the incubation. Pfus/ml were determined after 24 h at 37°C using standard phage plaque assay. The asterisks depict the statistical significance of the different samples when compared to the control with LB medium or water. For the samples treated with PMB, the statistical significance was analyzed in comparison to the respective sample without PMB. ns – not significant, ^∗∗^*p* < 0.01, ^∗∗∗^*p* < 0.001 and ^∗∗∗∗^*p* < 0.0001.

**FIGURE 8:**
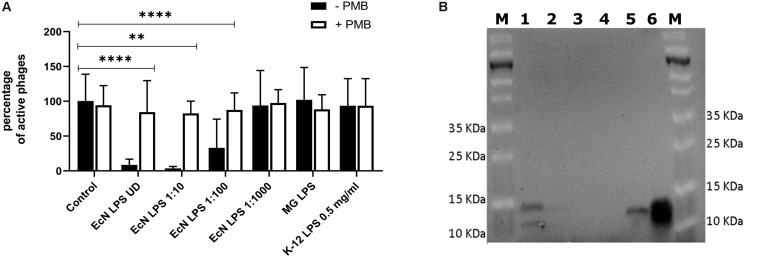
T4 phage inactivation by isolated LPS: 100 μl of LPS isolated from *E. coli* strains were diluted and were incubated with 100 μl of water (control) or T4 phages ± PMB for 1 h at 37°C and percentage of active phages were determined by the phage plaque assay **(A)**. ECN LPS UD – undiluted LPS isolated from EcN, EcN LPS 1:10 or 1:100 or 1:1000 – serially diluted EcN LPS isolations that were used in the coincubation, MG LPS – LPS undiluted from MG1655, K12 LPS 0.5 mg/ml – commercially available K-12 LPS (Cat no: tlrl-eklps, Invivogen). The asterisks depict the statistical significance of the different samples when compared to the control with water. ns – not significant, ^∗∗^*p* < 0.01 and ^∗∗∗∗^*p* < 0.0001. **(B)** The isolated LPS were visualized on 12% TruPAGE Precast Gels (Cat no: PCG2010-10EA, Sigma-Aldrich) and stained with Pro-QEmerald 300 LPS gel stain kit (Cat no: P20495, *Thermo Fisher* Scientific). [Lane description – M: Page Ruler Prestained Protein Ladder (Cat no: 26616, *Thermo Fisher* Scientific), 1: EcN LPS UD, 2: EcN LPS 1:10, 3: EcN LPS 1:100, 4: EcN LPS 1:1000, 5: MG1655 LPS, 6: K12 LPS 0.5 mg/ml].

**FIGURE 9:**
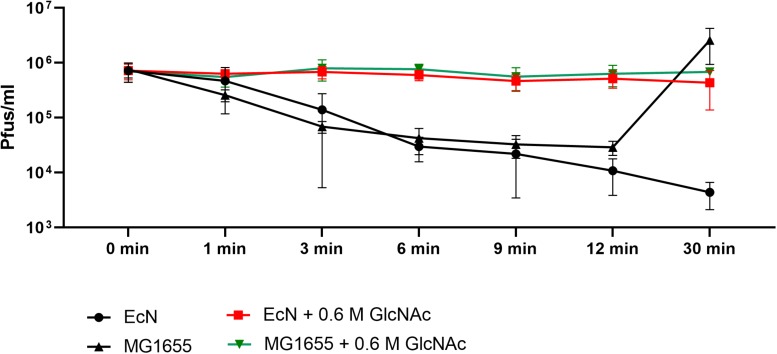
*N*-acetylglucosamine inhibits T4 phage adsorption to EcN: Graph depicts the kinetics of T4 phage titer reduction after the addition of T4 phages to the *E. coli* cultures in the presence and absence of 0.6 M *N*-acetylglucosamine (GlcNAc). T4 phages were added to mid-log phase (OD_600_∼0.5) EcN or MG1655 culture at an MOI of 0.01 ± 0.6 M GlcNAc, Pfus/ml were determined at different time points mentioned in the graph by phage plaque assay.

Polymyxin B (PMB) is an antibiotic that specifically binds to the LPS of gram-negative bacteria and it has been reported to destroy the LPS structure and thereby destroying its phage receptor activity (Koike and Iida, 1971). Hence, the role of EcN’s LPS in T4 phage inactivation was discerned by treating the supernatant of EcN, heat-killed EcN cells and supernatant of heat-killed cells with and without 25 μg/ml PMB for 1 h at 37°C followed by coincubation with T4 phages in presence or absence of 25 μg/ml PMB. The outcome of this study (Figure 7A) clearly displayed dramatic reduction or even complete loss of T4 phage inactivation ability of EcN samples upon treatment with 25 μg/ml PMB ranging from ∼150-fold less inactivation for EcN HK cells and complete loss of inactivation in case of EcN supernatant samples. Furthermore, treatment of EcN HK with higher concentrations of PMB also resulted in complete loss of T4 phage inactivation (Figure 7B). In addition, to further fathom the role of LPS in phage inactivation, LPS was isolated from EcN and MG1655 and was incubated with T4 phages, in presence and absence of 25 μg/ml PMB (Figure 8A). We noticed in this experimental setup that the UD and 1:10 diluted LPS isolated from EcN reduced the T4 phage titer by ∼85–90%. Furthermore, this function of LPS was absolutely abolished in the presence of 25 μg/ml PMB. It is well-known that LPS of *E. coli* K-12 strains is essential for the adsorption of T4 phages to their cells (Lindberg, 1973; Furukawa et al., 1979; Furukawa and Mizushima, 1982). But, as also reported previously (Mutoh et al., 1978; Beacham and Picken, 1981; Washizaki et al., 2016) we observed that isolated LPS from *E. coli* K-12 strain MG1655 and commercially purchased *E. coli* K-12 LPS failed to inactivate T4 phages (Figure 8A). In Figure 8B, the isolated LPS from different strains were visualized on SDS gel and the characteristic banding pattern for semi-rough type LPS of EcN was observed (Grozdanov et al., 2002) with a lower band referring to the lipid-A-core and the upper band representing single O6-antigen repeating unit. Whereas, in the case of K-12 strains only the core moiety was visible on the gel pointing out the remarkable difference between LPS of EcN and K-12 strains. In addition to the presence of O6-antigen, literature research also portrayed a distinct core oligosaccharide structure for EcN LPS (Supplementary Figure S5). The terminal component of the O-side chain of EcN’s LPS is *N*-acetylglucosamine (GlcNAc) and it is shown with *E. coli* B cells, that GlcNAc can inhibit the T4 phage adsorption by 100% (Dawes, 1975). If the binding of T4 phages to EcN’s LPS is responsible for inactivation of these phages, then the presence of GlcNAc should interfere not only with the binding of T4 and EcN’s LPS but also with the inactivation of T4 phages. To address this hypothesis, 0.6 M GlcNAc was added along with T4 phages to mid-log growing cultures of EcN and MG1655 cells (*E. coli*: T4 phage is 100:1) and Pfus/ml were determined after 1,3,6,9,12 and 30 min of phage addition. As shown in Figure 9, there was no reduction in T4 phage titer in the presence of 0.6 M GlcNAc. Whereas in the absence of GlcNAc, there was a gradual decrease in T4 phage titer when incubated with EcN cells of up to ∼100-fold after 30 min of incubation which indicates the absorption of T4 phages to EcN. In contrary to EcN, an increase in T4 phage titer was noticed in the absence of GlcNAc after 12 min of incubation with MG1655 cells indicating a successful infection and phage propagation.

### EcN Inhibits T4 Phage Infection of *E. coli* K-12 Strains

**FIGURE 10:**
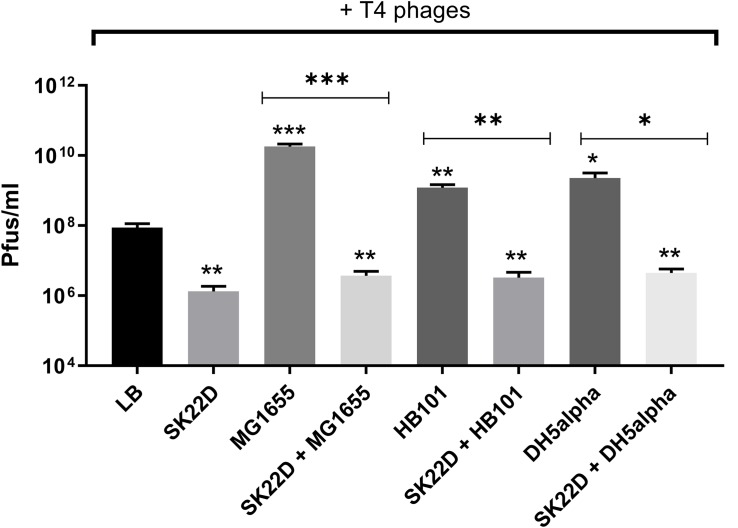
Interference by EcN on T4 phage infection of K-12 strains: T4 phages were incubated in mono; co; or tri-cultures with the microcin negative EcN strain SK22D and/or the K-12 strains MG1655 or HB101 or DH5α (MOI of 1:1:1) at 37°C for 24 h, static and Pfus/ml were determined by phage plaque assay. LB: control (T4 phages + LB medium). The asterisks on the bar depict the statistical significance of the different samples when compared to the control with LB medium and the asterisks on the lines depict the statistical significance of different cocultures when compared to their respective tri-cultures. ^∗^*p* < 0.05, ^∗∗^*p* < 0.01, and ^∗∗∗^*p* < 0.001.

Since EcN was shown in this study to inactivate T4 phages we queried whether this EcN property might also result in protection of *E. coli* K-12 strains against T4 phage infection. To address this question, T4 phages were incubated with *E. coli* K-12 strains (MG1655, DH5α and HB101) in the presence or absence of SK22D (a microcin negative mutant of EcN) at 37°C for 24 h and Pfus/ml were determined. SK22D was employed in this experiment to avoid killing of the K-12 strains by the two microcins produced by wildtype EcN (Patzer et al., 2003). As expected, all three tested K-12 strains (MG1655, DH5α, HB101) were infected by T4 phages resulting in a ∼100 (MG1655) or 50-fold (DH5a, HB101) increase in phage titer (Figure 10). In contrast, the presence of SK22D resulted in a phage titer reduction of ∼10,000-fold compared to MG1655 alone and ∼1000-fold less Pfus/ml or DH5a and HB101 in the presence of SK22D compared to the respective K-12 strain without SK22D (Figure 10). Hence, our results exhibited that EcN interferes with T4 phage infection of the tested K-12 strains very efficiently.

## Discussion

Bacteria-phage interactions and subsequent evolution is a fascinating research focus and in certain cases, these interaction leads to the emergence of new gastrointestinal pathogens which generally exhibit enhanced virulence and antibiotic resistance (Bielaszewska et al., 2011). On the other hand, bacterial strains have evolved to modify their surface structures to counteract these phage infections (Nordstrom and Forsgren, 1974; Reyes-Cortes et al., 2012; Denes et al., 2015). Owing to the clinical significance of EcN (Rozanska et al., 2014; Scaldaferri et al., 2016; Sonnenborn, 2016) and it’s observed anti-shiga toxin effect against EHEC strains (Reissbrodt et al., 2009; Rund et al., 2013), it is of high priority to understand the phage sensitivity of EcN prior to its prescription in the treatment of EHEC infections. Our group has previously investigated the lambdoid phage resistance of EcN and reported that EcN was not infected by the tested lysogenic lambdoid phages: *stx*-phages, lambda-phages (Bury et al., 2018). However, in case of interaction with lytic phages, there is an entirely different scenario, in which the bacteria are lysed upon infection. Extended survival and efficient colonization of the gut are the preferred characteristics for a probiotic strain and hence phage-mediated lysis would be a big threat for any strain used in the prophylaxis and/or treatment. For this reason, in this study, we focused on understanding the sensitivity of EcN toward the widely studied and well understood lytic T4 phage. Initial experiments with T4 phages involving PPAs and liquid culture assays (Figure 1) clearly demonstrated the T4 phage resistance of EcN. It was further validated by microscopic experiments, where the micrographs (Figures 2, 3A) displayed intact viable EcN cells, in spite, the T4 phages were attached to their cell surface during coincubation.

To sustain in a complex environment such as the gut and to combat the interminable phage attacks, bacteria must possess a lot of fitness factors contributing to the phage resistance and these factors are usually the result of phage-bacterial coevolution (Mirzaei and Maurice, 2017; De Sordi et al., 2019). In the present study, we focused to identify the factor(s) of EcN involved in T4 phage resistance. From our results, we noticed that, despite insensitivity of EcN, the T4 phages were found to be well associated with the EcN cells during incubation (Figure 3) and hence we assumed the candidate responsible for resistance is a cell surface component or structure of EcN. Different surface mutants of EcN [curli biofilm (*csg*), flagellin (*fliC*), cellulose (*bcs*)] were tested (data not shown) for their sensitivity with T4 phages. However, only with the capsule negative EcN mutant, plaque assay results demonstrated T4 infection (Figure 4), highlighting the importance of the K5 capsule in T4 phage resistance. Capsule mediated phage-resistance was described previously in other bacteria (Kauffmann and Vahlne, 1945; Sapelli and Goebel, 1964; Scholl et al., 2005; Majkowska-Skrobek et al., 2018) and is a widely reported phage resistance mechanism by masking the phage receptor and thus preventing phage adsorption (Labrie et al., 2010). However, our microscopic experiments and PCR with EcN wildtype clearly showed phages attached to EcN. Hence, there must be another moiety at the surface to which the T4 phages are getting attached, however, are not being able to subsequently infect EcN.

Bacterial phage resistance mechanisms can be broadly classified into two categories based on their mode of protection: one being self-protection, which includes strategies like (i) surface structure modifications e.g., phage receptor mutation, cell wall modification, etc. (Sijtsma et al., 1990; Chatterjee and Rothenberg, 2012; Kim and Ryu, 2012; Leon and Bastias, 2015; Li et al., 2018) (ii) genome modification that leads to blockage of incoming phage DNA e.g., superinfection exclusion systems (Hofer et al., 1995; Cumby et al., 2012) (iii) CRISPR-Cas or restriction-modification systems that degrades the incoming phage DNA (Arber et al., 1963; Gasiunas et al., 2014; Chakraborty et al., 2019). However, there is another interesting mode that is being extensively studied in the recent past: inactivation of the phages in the bacterial environment by binding to surface structures of outer membrane vesicles or to the extracellular matrix of bacterial e.g., biofilms (Manning and Kuehn, 2011; Abedon, 2017; Vidakovic et al., 2018). Correspondingly, our data very well revealed that in addition to self-protection, EcN also inactivates the T4 phages in coincubation experiments (Figure 5). The fact that both the cells and cell-free supernatant of EcN were able to perform this effect, led to the hypothesis that material which is not the only cell-attached, but also present in the supernatant might be responsible for inactivation of T4 phages. Noteworthy, the supernatant of the EcN Δ*K5* strain also inactivated the phages (Supplementary Figure S3) reassuring our hypothesis on the participation of more than one defense associated factor in EcN. Biochemical experiments involving PMB treatment abolished the T4 phage inactivation ability of EcN and EcN Δ*K5* supernatant samples and experiments with isolated LPS, showcased the ability of EcN’s LPS in T4 phage inactivation (Figure 8 and Supplementary Figure S3).

We also tested the T4 phage inactivation ability of other *E. coli* strains (Supplementary Figure S4) and only the uropathogenic strain CFT073 which harbors the same O antigen type as EcN (i.e., O6 type LPS) inactivated the T4 phages as efficiently as EcN. In contrast, both the cells and the supernatant of the commensal *E. coli* strains SE11 and SE15 harboring LPS of other O-antigen types did not inactivate the T4 phages (Supplementary Figures S4, S5B). To summarize our observation, the K-12 strains that lack O-antigen were infected by T4 phages, commensal strains that have non-O6 type LPS were not able to inactivate the T4 phages and only the strains EcN and CFT073 that harbor O6 type LPS inactivated the T4 phages. From these results, we could conclude that T4 phage inactivation by LPS is O-antigen-specific. Nevertheless, further experiments involving O6 antigen deletion mutants are required to validate this hypothesis. Until now, the semi-rough LPS of EcN resulting from a single nucleotide mutation in the O-antigen polymerase gene is known to be responsible for EcN’s serum sensitivity (Grozdanov et al., 2002) while in this study, we have disclosed a new function for EcN’s LPS: “T4 phage inactivation”. With an O6 antigen and R1 type core oligosaccharide, EcN’s LPS is very different from K-12 LPS (Supplementary Figure S5A). The distal end of O6 antigen in EcN’s LPS is composed of *N*-acetylglucosamine (GlcNAc) (Supplementary Figure S5B) and hence hypothesized to be the interacting partner with the T4 phages. This hypothesis was validated by our results which displayed absolute inhibition of T4 phage binding to EcN in the presence of 0.6 M GlcNAc. Moreover, in *E. coli* B cells, in addition to GlcNAc, other monosaccharides like glucosamine, 2-deoxyglucose, 3-O-methyl glucose, and gluconolactone inhibited T4 phage adsorption (Dawes, 1975). In contrary, glucose did not inhibit the phage adsorption (data not shown) to EcN and hence we observed a very specific GlcNAc mediated inhibition of T4 phage adsorption to EcN cells suggesting GlcNAc at the distal end of O6 antigen to be the interacting partner with T4 phages. Furthermore, with our results, we can assume that most likely EcN sequestered the phages in the environment with its LPS and thereby not only protecting itself but also protecting the otherwise sensitive bacteria in the environment. Our hypothesis was confirmed by our experiments with the tri-culture set up which proved the protection of K-12 strains against T4 phage infection by EcN (Figure 10). Nevertheless, extensive research with experiments focusing on the interaction between different players in the tri-culture set up must be done to shed more light on the mechanism of protection.

## Conclusion

In conclusion, in this study, we have uncovered EcN’s resistance toward lytic T4 phages, specifically highlighting the role of two of its surface structures in phage defense. First, the K5 polysaccharide capsule which protects EcN from T4 phage infection. Secondly, we demonstrated for the first time the LPS mediated T4 phage inactivation by EcN without disintegration of the phage particles. These properties (among others) are most likely important for EcN’s survival in the gut and for its ability to execute protective effects in the framework of its probiotic nature.

## Data Availability Statement

The RNA-Seq data presented in this work can be accessed through GEO series accession number: GSE135946 (https: //www.ncbi.nlm.nih.gov/geo/query/acc.cgi?acc=GSE135946).

## Author Contributions

TO and RB conceived and designed the experiments. MS performed the experiments. MS and TO analyzed the data and manuscript preparation. MS, RB, and TO manuscript revision. All authors read and approved the final version of the manuscript.

## Conflict of Interest

The study was supported by a stipend to MS by the company Pharma-Zentrale GmbH, Germany. The remaining authors declare that this study received funding from Pharma-Zentrale GmbH and Ardeypharm GmbH. The funders had the following involvement with the study: study design, the decision to submit this article for publication.

## Supplementary Material

FIGURE S1 Genotypic verification of EcN capsule mutant by PCR: For 10 single colonies of EcN Δ*k5*, a verification PCR was performed with primer pair K5_1 and 2 (left), K5_3 and 4 (right) using 2× PCR MM as described in the “Materials and Methods” section. Lane description: 1–10: EcN Δ*k5* colonies; 11: EcN wildtype; N: water control; M – GeneRuler 100 bp DNA Ladder (Cat no: SM0241, Thermo Scientific). (Expected amplicon size with K5_1 and 2: for EcN wildtype – No amplicon and for EcN Δ*k5* – 1083 bp and for K5_3 and 4: EcN wildtype – 1045 bp, EcN Δ*k5* – No amplicon).

FIGURE S2 The Donut graphs represents the functional grouping of the genes that were downregulated **(i)** and upregulated **(ii)** in EcN when incubated with T4 phages.

FIGURE S3 T4 phage inactivation by EcN capsule negative mutant: The graph shows the T4 phage titers in Pfus/ml after 24 h of coincubation with EcN or EcN Δ*k5* samples. Samples were processed as described in the table in Figure 5C. Further, some samples were treated with 25 μg/ml PMB to test the role of LPS of the supernatant of EcN Δ*k5* in T4 phage inactivation. The asterisks on the bar depict the statistical significance of the different samples when compared to the control with LB medium. ns – not significant, ^∗^*p* < 0.05, ^∗∗^*p* < 0.01.

FIGURE S4 T4 phage inactivation by other *E. coli* strains and their supernatant: Graphs here displayed the result of coincubation studies performed with *E. coli* cells **(A)** and their 10× concentrated supernatant **(B)** that was prepared as described in the “Materials and Methods” section. The processed samples were incubated with T4 phages for 24 h at 37°C, static after which the samples were sterile filtered, and Pfus/ml were determined by phage plaque assay. The asterisks on the bar depict the statistical significance of the different samples when compared to the control with LB medium. ns – not significant, ^∗∗^*p* < 0.01, ^∗∗∗^*p* < 0.001.

FIGURE S5 Comparison of LPS among different *E. coli* strains used in the study: Structural comparison of LPS from EcN and *E. coli* K-12 strain **(A)**. The dotted line in the K-12 core between GlcN and Hep indicates GlcN being present only in some *E. coli* K-12 strains (Orskov et al., 1977). The O antigen structures of different *E. coli* strains used in this study were listed out **(B)**. The abbreviations used in this figure are GlcN: N-acetylglucosamine, Man: mannose, Glu: glucose, Gal: galactose, Hep: L-glycero-D-manno heptose, Kdo: 3-deoxy-D-manno-oct- 2ulosonic acid, P: phosphate, Gal: N-acetylgalactosamine, Rhap: rhamno pyranose, GlcN(Slac): 2-acetamido-4-O-[(S)-1-carboxy-ethyl]-2-deoxy-D-glucose.

TABLE S1 Details of gene regulation in EcN incubated with T4 phages after 2 h when compared to EcN incubated in LB medium for 2 h.

TABLE S2 Details of the genes downregulated in EcN when incubated with T4 phages after 2 h (log2foldchange < -2, padj < 0.05).

TABLE S3 Details of the genes upregulated in EcN when incubated with T4 phages after 2 h (log2foldchange > 2, padj < 0.05).

DATA SHEET S1 Transcriptomic response of EcN to T4 phages after 2 h of incubation: RNA was isolated from EcN incubated with and without T4 phages for 2 h in LB medium, deep-sequenced and transcriptome were further compared and analyzed. 2.7% of the total genes were differentially regulated. In this data sheet, results of transcriptome analysis are presented as Supplementary Tables S1–S3 and Supplementary Figure S2. The RNA-Seq data presented in this work can be accessed through GEO series accession number: GSE135946 (https://www.ncbi.nlm.nih.gov/geo/query/acc.cgi?acc=GSE135946).
