# Supplementary figures and images for: K5 Capsule and Lipopolysaccharide Are Important in Resistance to T4 Phage Attack in Probiotic E. coli Strain Nissle 1917
Source: Front Microbiol. 2019 Nov 29;10:2783. doi: 10.3389/fmicb.2019.02783 (PMC6895014; doi:10.3389/fmicb.2019.02783)

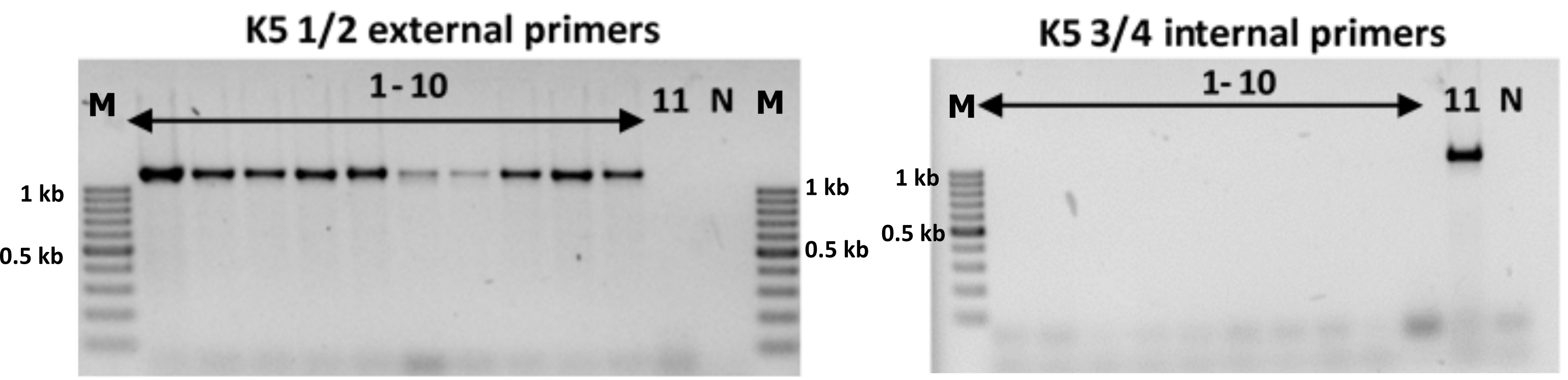

Supplement: FIGURE S1 — Genotypic verification of EcN capsule mutant by PCR: For 10 single colonies of EcN Δk5, a verification PCR was performed with primer pair K5_1 and 2 (left), K5_3 and 4 (right) using 2× PCR MM as described in the “Materials and Methods” section. Lane description: 1–10: EcN Δk5 colonies; 11: EcN wildtype; N: water control; M – GeneRuler 100 bp DNA Ladder (Cat no: SM0241, Thermo Scientific). (Expected amplicon size with K5_1 and 2: for EcN wildtype – No amplicon and for EcN Δk5 – 1083 bp and for K5_3 and 4: EcN wildtype – 1045 bp, EcN Δk5 – No amplicon). [file Image_1.TIFF]

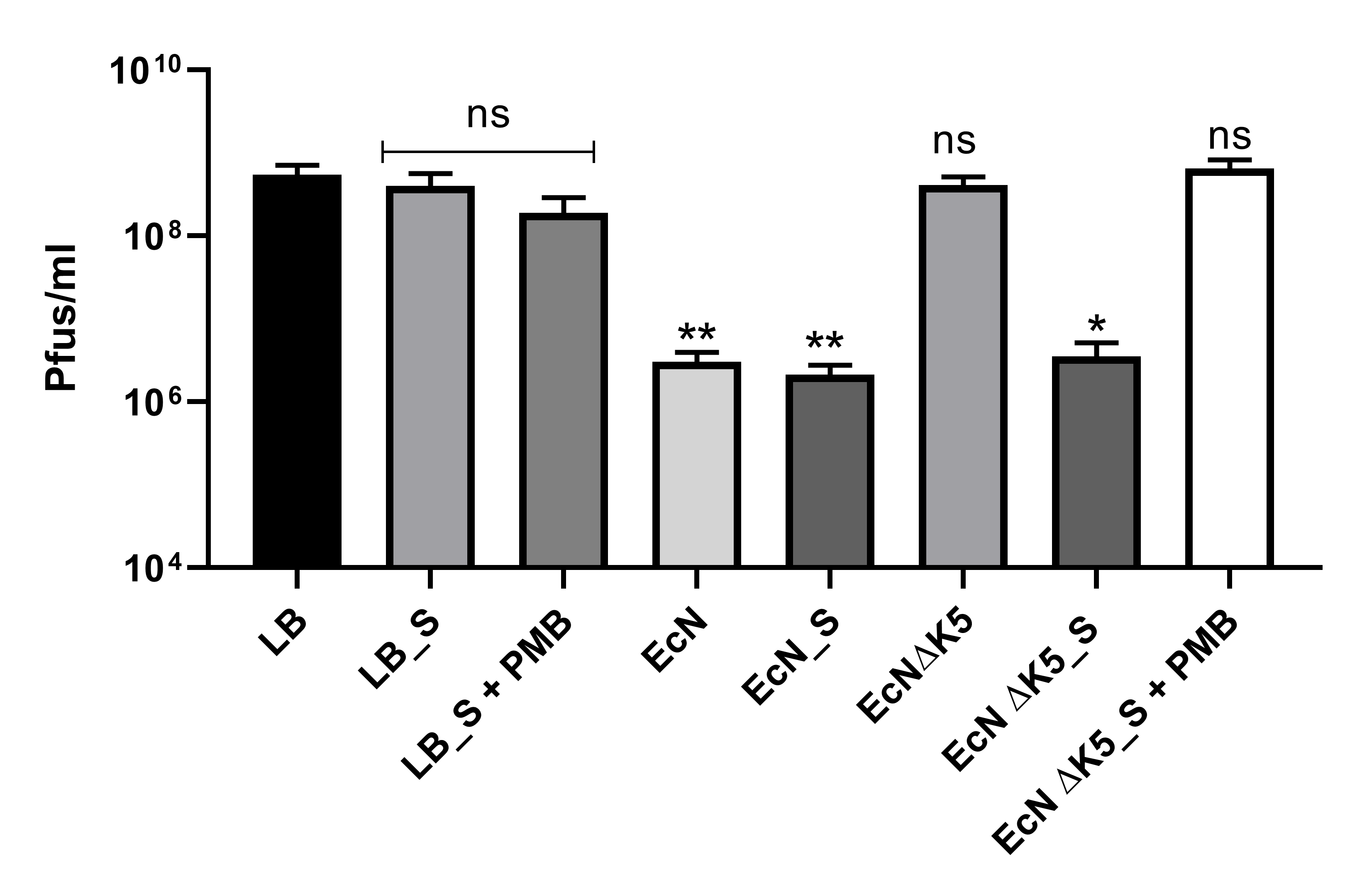

Supplement: FIGURE S3 — T4 phage inactivation by EcN capsule negative mutant: The graph shows the T4 phage titers in Pfus/ml after 24 h of coincubation with EcN or EcN Δk5 samples. Samples were processed as described in the table in Figure 5C. Further, some samples were treated with 25 μg/ml PMB to test the role of LPS of the supernatant of EcN Δk5 in T4 phage inactivation. The asterisks on the bar depict the statistical significance of the different samples when compared to the control with LB medium. ns – not significant, ∗p < 0.05, ∗∗p < 0.01. [file Image_3.TIF]

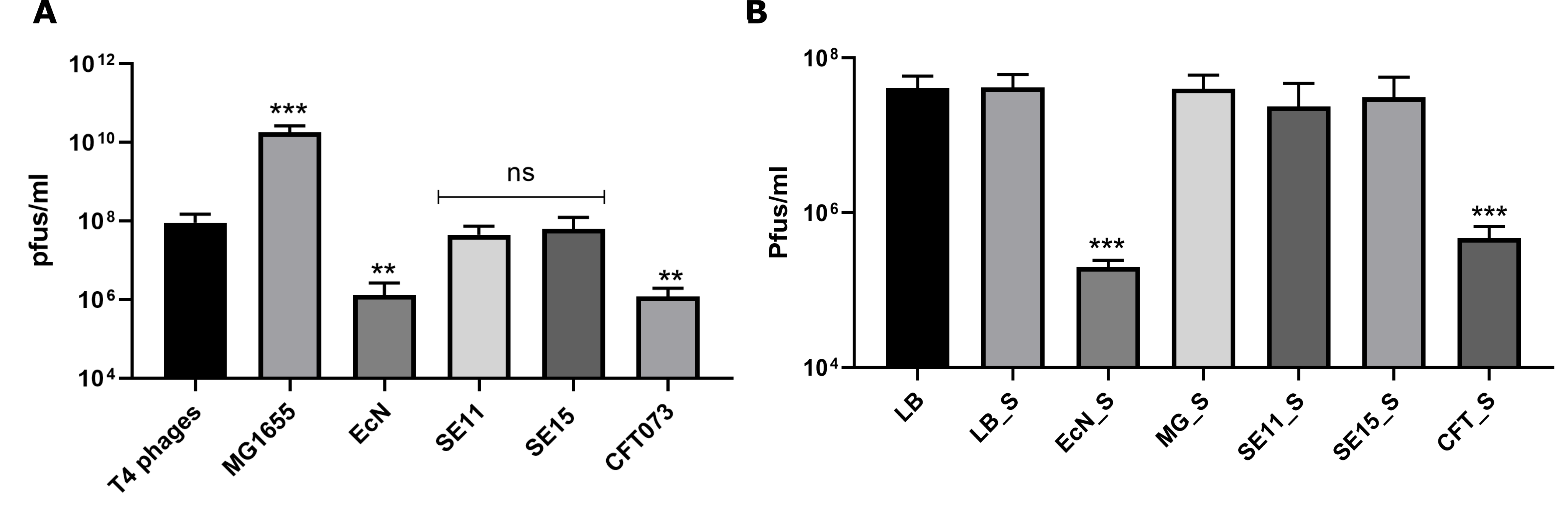

Supplement: FIGURE S4 — T4 phage inactivation by other E. coli strains and their supernatant: Graphs here displayed the result of coincubation studies performed with E. coli cells (A) and their 10× concentrated supernatant (B) that was prepared as described in the “Materials and Methods” section. The processed samples were incubated with T4 phages for 24 h at 37°C, static after which the samples were sterile filtered, and Pfus/ml were determined by phage plaque assay. The asterisks on the bar depict the statistical significance of the different samples when compared to the control with LB medium. ns – not significant, ∗∗p < 0.01, ∗∗∗p < 0.001. [file Image_4.TIFF]
