# Supplementary material for: K5 Capsule and Lipopolysaccharide Are Important in Resistance to T4 Phage Attack in Probiotic E. coli Strain Nissle 1917
Source: Front Microbiol. 2019 Nov 29;10:2783. doi: 10.3389/fmicb.2019.02783 (PMC6895014; doi:10.3389/fmicb.2019.02783)
Supplement: FIGURE S2 — The Donut graphs represents the functional grouping of the genes that were downregulated (i) and upregulated (ii) in EcN when incubated with T4 phages. [file Data_Sheet_1.pdf]

Transcriptome analysis of EcN incubated with T4 phages for 2 h

Material and methods

Extracted RNA was depleted of ribosomal RNA using the Ribo-Zero rRNA Removal Kit for bacteria (Illumina) according to the manual. Depleted RNA was fragmented for 3 min at 94°C using the NEBNext Magnesium RNA Fragmentation Module. The RNA ends were repaired with two consecutive T4 PNK incubations (-/+ ATP) and a RppH treatment. Library preparation was performed according to the NEBNext Multiplex Small RNA Library Preparation Guide for Illumina. All adapters and primers were diluted 1:4 and 15 and 16 cycles of PCR were used, respectively. No size selection was performed at the end of the protocol. 12 libraries were pooled and sequenced on a NextSeq 500 with a read length of 75 nt.

Analysis of deep-sequencing data

The quality of raw reads (Phred scores, number of duplicates and adapter) were assessed using FastQC (version-0.11.31) (Andrews, 2010). In order to assure a high sequence quality, the Illumina reads in FASTQ format were trimmed with a cut-off phred score of 20 by cutadapt (version-1.15) (Martin, 2011) that also was used to remove the sequencing adapter sequences. The following steps were performed using the subcommand "create", "align" and "coverage" of the tool READemption (version-0.4.3)(Forstner et al., 2014) with default parameters. Reads with a length below 20nt were removed and the remaining reads were mapped to the reference genome sequences (NCBI Reference Sequence: NZ_CP007799.1, [27 June 2014]) using segemehl (Hoffmann et al., 2009). Coverage plots in wiggle format representing the number of aligned reads per nucleotide were generated based on the aligned reads and visualized in the Integrated Genome Browser (Freese et al., 2016). Each graph was normalized to the total number of reads that could be aligned from the respective library. To restore the original data range and prevent rounding of small error to zero by genome browsers, each graph was then multiplied by the minimum number of mapped reads calculated over all libraries. The differentially expressed genes were identified using DESeq2 (version-1.16.1)(Love et al., 2014). In all cases, only genes with maximum Benjamini-Hochberg corrected p-value (padj) of 0.05, were classified as significantly differentially expressed. The data were represented as MA plots using R. The RNA-Seq data presented in this work has been deposited at the NCBI Gene Expression Omnibus (Edgar et al., 2002) and can be accessed through GEO series accession number GSE135946 (https://www.ncbi.nlm.nih.gov/geo/query/acc.cgi?acc=GSE135946).

The results of the transcriptome analysis are presented here in the sections table S1, figure S2, table S2 and table S3

Table S1: Details of gene regulation in EcN incubated with T4 phages after 2 h when compared to EcN incubated in LB medium for 2 h

Genes downregulated (log_2foldchange<-2, padj<0.05)	123 genes
Genes upregulated (log_2foldchange>2, padj<0.05)	33 genes

Figure S2: Donut graphs representing the functional grouping of the genes that were downregulated (i) with log2foldchange<-2, padj<0.05 and upregulated (ii) with log2foldchange>2, padj<0.05 in EcN when incubated with T4 phages after 2 h. In the legend of the graph, the numbers in the bracket after the functional groups represents number of genes down or upregulated in the respective group.

i)

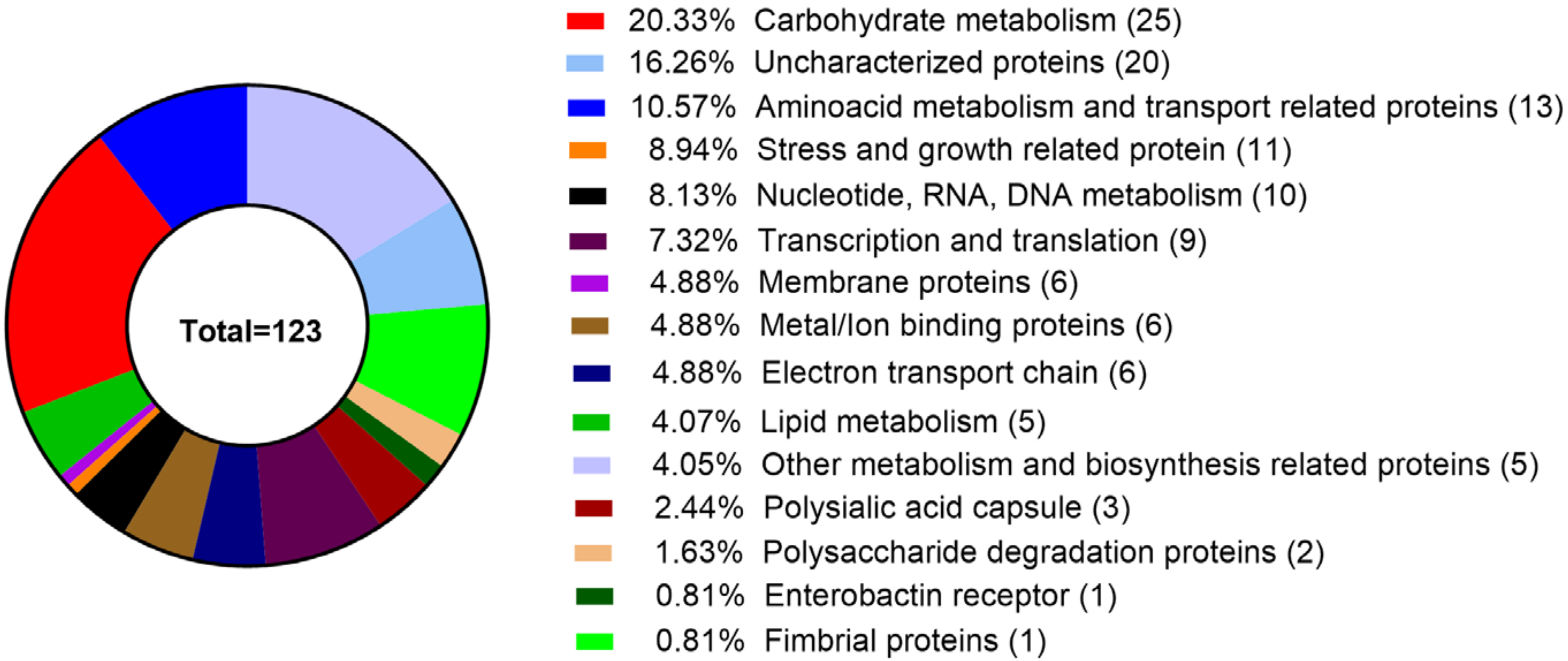

ii)

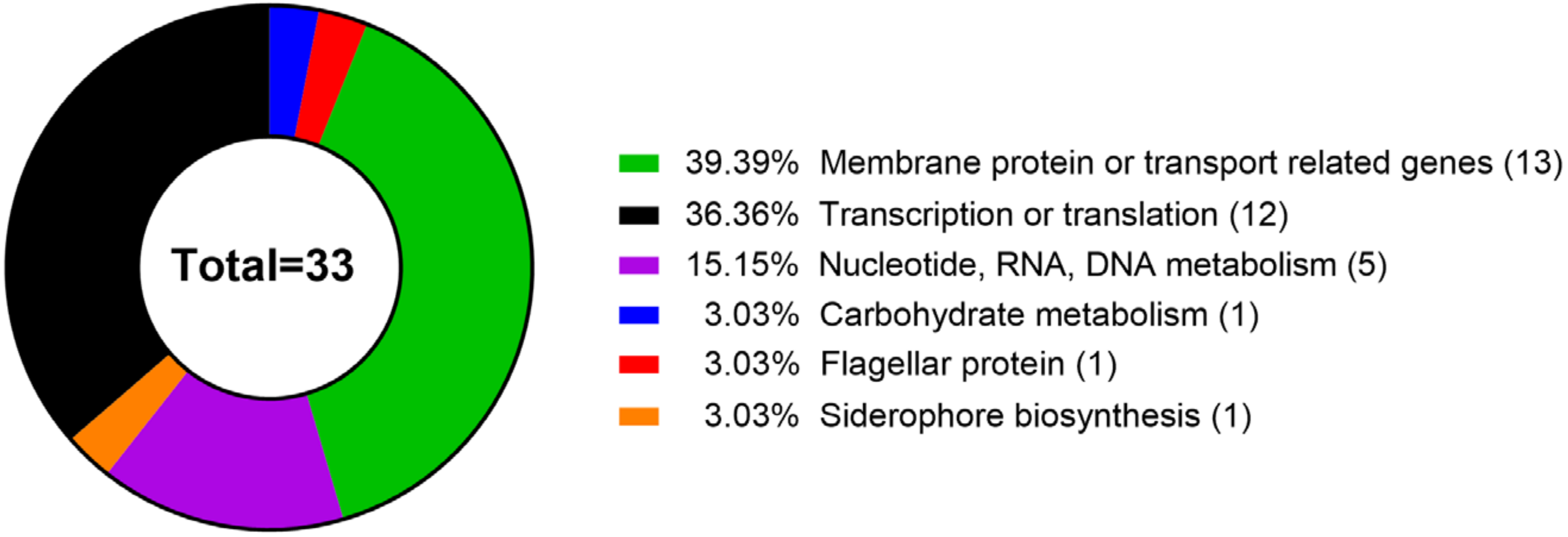

Table S2: Details of the genes downregulated in EcN when incubated with T4 phages after 2 h (log_2foldchange<-2, padj<0.05)

Downregulated genes	log_2foldchange	Functional prediction	Functional group
**EcN1917_4157**	-5,074	Tryptophan production	Aminoacid metabolism and transport related
**EcN1917_3127**	-5,035	Aspartate/ornithine carbamoyl transferase family protein protein YgeW,	Nucleotide, RNA, DNA metabolism
**EcN1917_1644**	-4,859	N-acetylgalactosamine 6-sulfate sulfatase (GALNS), polysaccharide degradation protein	Polysaccharide degradation proteins
**EcN1917_4791**	-4,850	Uncharachetrised protein	Uncharachetrised proteins
**EcN1917_4158**	-4,557	Tryptophan-specific transport protein	Aminoacid metabolism and transport related genes

**EcN1917_4896**	-4,312	N-acetylneuraminic acid outer membrane channel protein NanC	Membrane proteins
**EcN1917_3113**	-4,098	Diaminopimelate decarboxylase (EC 4.1.1.20), amino acid biosynthesis	Aminoacid metabolism and transport related genes
**EcN1917_3447**	-4,033	Glycogen synthesis protein GlgS , glycogen synthesis	Carbohydrate metabolism
**EcN1917_3135**	-3,953	Predicted oxidoreductase 2C Fe-S subunit, membrane proteins	Membrane proteins
**EcN1917_3128**	-3,945	Diaminopropionate ammonia-lyase (EC 4.3.1.15), amino acid metabolsim	Aminoacid metabolism and transport related genes
**EcN1917_4920**	-3,898	Uncharachetrised protein	Uncharachetrised proteins
**EcN1917_4483**	-3,787	Zinc resistance associated protein, stress related protein	Stress related proteins
**EcN1917_2521**	-3,762	Acetyl-CoA:acetoacetyl-CoA transferase 2C alpha subunit (EC 2.8.3.8), lipid/fatty acid metabolism	Lipid metabolism
**EcN1917_3136**	-3,752	SsnA protein, metal/ion binding	Metal/ion binding
**EcN1917_3523**	-3,710	Uncharachetrised protein	Uncharachetrised proteins
**EcN1917_2441**	-3,598	Pseudouridine 5'-phosphate glycosidase, nucleotide metabolism	Nucleotide, RNA, DNA metabolism
**EcN1917_1574**	-3,590	YdcH protein , uncharachterised protein	Uncharachetrised proteins
**EcN1917_0001**	-3,586	Aspartokinase (EC 2.7.2.4) / homoserine dehydrogenase (EC 1.1.1.3), homoserine biosynthetic process	Aminoacid metabolism and transport related genes
**EcN1917_2442**	-3,512	Pseudouridine kinase (EC 2.7.1.83), nucleotide metabolism	Nucleotide, RNA, DNA metabolism
**EcN1917_3129**	-3,436	Putative deacetylase YgeY, metal/ion binding	Metal/ion binding
**EcN1917_2543**	-3,428	Uncharachetrised protein	Uncharachterised protein

**EcN1917_3133**	-3,386	Accessory protein YqeC in selenium-dependent molybdenum hydroxylase maturation, transcription and translation	Transcription and translation
**EcN1917_3137**	-3,344	Uncharacterised protein YgfM	Uncharachterised protein
**EcN1917_2868**	-3,250	Ribosome hibernation protein YfiA, translation	Transcription and translation
**EcN1917_3072**	-3,210	Type 6 secretion system tube protein	Membrane proteins
**EcN1917_2542**	-3,175	Anaerobic glycerol-3-phosphate dehydrogenase subunit C (EC 1.1.5.3), electron transport chain	Electron transport chain
**EcN1917_4488**	-3,074	Translation elongation factor Tu	Transcription and translation
**EcN1917_4482**	-3,045	Zinc resistance-associated protein, stress related protein	Stress related proteins
**EcN1917_4723**	-3,040	Possible membrane protein	Membrane proteins
**EcN1917_1544**	-3,032	Universal stress protein F	Stress related proteins
**EcN1917_2652**	-3,012	D-serine permease DsdX, D-serine transport	Aminoacid metabolism and transport related genes
**EcN1917_1383**	-2,981	Uncharacterised protein	Uncharachterised protein
**EcN1917_0003**	-2,971	Threonine synthase (EC 4.2.3.1), threonine biosynthetic process	Aminoacid metabolism and transport related genes
**EcN1917_3134**	-2,966	CTP:molybdopterin cytidylyltransferase , translation	Transcription and translation
**EcN1917_2538**	-2,940	Glycerophosphoryl diester phosphodiesterase 2C periplasmic, lipid metabolism	Lipid metabolism
**EcN1917_4895**	-2,931	Sialic acid-induced transmembrane protein YjhT(NanM) 2C possible mutarotase, polysialicacid capsule metabolism	Polysialicacid capsule
**EcN1917_2426**	-2,924	Galactose/methyl galactoside ABC transport system 2C D-galactose-binding periplasmic protein MglB (TC 3.A.1.2.3), galactose transport	Carbohydrate metabolism

**EcN1917_2540**	-2,904	Anaerobic glycerol-3-phosphate dehydrogenase subunit A (EC 1.1.5.3), lipid metabolism	Lipid metabolism
**EcN1917_3610**	-2,898	Putative sugar isomerase involved in processing of exogenous sialic acid, polysialicacid capsule metabolim	Polysialicacid capsule
**EcN1917_2423**	-2,891	Uncharacterised protein	Uncharachterised protein
**EcN1917_0906**	-2,883	Cold shock protein CspD	Stress related protein
**EcN1917_1643**	-2,881	GALNS arylsulfatase regulator (Fe-S oxidoreductase), metal/ion binding	Metal/ion binding
**EcN1917_3138**	-2,857	Possible hypoxanthine oxidase XdhD, purine nucleobase metabolic process	Nucleotide, RNA, DNA metabolism
**EcN1917_0002**	-2,839	Homoserine kinase (EC 2.7.1.39), threonine biosynthetic process	Aminoacid metabolism and transport related genes
**EcN1917_0616**	-2,808	Enterobactin receptor protein	Enterobactin receptor
**EcN1917_0651**	-2,795	Citrate [pro-3S]-lyase] ligase (EC 6.2.1.22), involved in biosynthetic process	Other metabolism and biosynthesis related proteins
**EcN1917_3664**	-2,784	Uncharacterised protein	Uncharachterised protein
**EcN1917_4722**	-2,772	Putative Uncharacterised protein YjfN	Uncharachterised protein
**EcN1917_4790**	-2,735	Arginine deiminase (EC 3.5.3.6), arginine metabolic process	Aminoacid metabolism and transport related genes
**EcN1917_1048**	-2,730	Galactosamine-6-phosphate isomerase, glycolysis	Carbohydrate metabolism and transport related genes
**EcN1917_2440**	-2,729	Inner membrane transport protein YeiM 2C in cluster with pseudouridine metabolism operon, RNA metabolism	Nucleotide, RNA, DNA metabolism
**EcN1917_1014**	-2,658	Uncharacterised protein	Uncharachterised protein

**EcN1917_4789**	-2,634	Carbamate kinase (EC 2.7.2.2), arginine metabolic process	Aminoacid metabolism and transport related genes
**EcN1917_2539**	-2,629	Glycerol-3-phosphate transporter, glycolysis	Carbohydrate metabolism and transport related genes
**EcN1917_2541**	-2,624	Anaerobic glycerol-3-phosphate dehydrogenase subunit B (EC 1.1.5.3), lipid metabolism	Lipid metabolism
**EcN1917_0062**	-2,617	L-ribulose-5-phosphate 4-epimerase (EC 5.1.3.4), carbohydrate metabolism	Carbohydrate metabolism and transport related genes
**EcN1917_2421**	-2,616	Pyridine nucleotide-disulphide oxidoreductase family protein, electron transport chain	Electron transport chain
**EcN1917_0407**	-2,593	Threonine efflux protein,	Aminoacid metabolism and transport related genes
**EcN1917_0417**	-2,592	Galactoside O-acetyltransferase, lactose biosynthesis	Carbohydrate metabolism
**EcN1917_0035**	-2,577	Transcriptional activatory protein CaiF, transcription	Transcription and translation
**EcN1917_2813**	-2,577	Stationary phase inducible protein CsiE, stress/ growth related protein	Stress related protein
**EcN1917_4398**	-2,554	Glycerol uptake facilitator protein, glycerol transport	Carbohydrate metabolism
**EcN1917_2653**	-2,552	D-serine dehydratase (EC 4.3.1.18), D-serine metabolism	Aminoacid metabolism and transport related genes
**EcN1917_2422**	-2,528	Dihydropyrimidine dehydrogenase [NADP+] (EC 1.3.1.2), nucleotide metabolism	Nucleotide, RNA, DNA metabolism
**EcN1917_2537**	-2,521	Protein inaA, stress related protein	Stress related protein
**EcN1917_0753**	-2,518	Uncharacterised protein	Uncharachterised protein
**EcN1917_4134**	-2,512	Heat shock protein, stress related protein	Stress related protein
**EcN1917_0275**	-2,483	Uncharacterised protein	Uncharachterised protein

**EcN1917_2958**	-2,479	tRNA metabolism, translation	Transcription and translation
**EcN1917_3520**	-2,474	D-glucarate catabolic process	Carbohydrate metabolism
**EcN1917_3521**	-2,464	2-hydroxy-3-oxopropionate reductase (EC 1.1.1.60), glyoxylate metabolic process	Carbohydrate metabolism
**EcN1917_3132**	-2,446	Selenium-dependent molybdenum hydroxylase system protein YqeB, transcription	Transcription and translation
**EcN1917_3019**	-2,445	Uncharacterised protein	Uncharachterised protein
**EcN1917_3517**	-2,419	Threonine catabolic operon transcriptional activator TdcA, transcription	Transcription and translation
**EcN1917_0631**	-2,417	Carbon starvation protein A, stress related protein	Stress related protein
**EcN1917_2252**	-2,414	Uncharacterised protein	Uncharachterised protein
**EcN1917_2519**	-2,398	Sensory histidine kinase AtoS, membrane protein	Membrane protein
**EcN1917_1013**	-2,392	Glucose-1-phosphatase (EC 3.1.3.10), glycolysis	Carbohydrate metabolism and transport related genes
**EcN1917_0063**	-2,374	L-arabinose isomerase (EC 5.3.1.4), carbohydrate metabolism a	Carbohydrate metabolism and transport related genes
**EcN1917_0675**	-2,373	Uncharacterised protein	Uncharachterised protein
**EcN1917_4649**	-2,349	Melibiose operon regulatory protein, transcription	Transcription and translation
**EcN1917_2271**	-2,340	Uncharacterised protein	Uncharachterised protein
**EcN1917_2741**	-2,337	Ethanolamine utilization polyhedral-body-like protein EutS, polysaccharide degradation	Polysaccharide degradation proteins
**EcN1917_3131**	-2,320	Carbamate kinase-like protein YqeA	Aminoacid metabolism and transport related genes
**EcN1917_4397**	-2,314	Glycerol kinase (EC 2.7.1.30)	Lipid metabolism
**EcN1917_1015**	-2,307	Flavoprotein WrbA, stress related protein	Stress related protein

**EcN1917_3613**	-2,306	Sialic acid transporter (permease) NanT, polysialicacid capsule metabolism	Polysialicacid capsule metabolism
**EcN1917_1387**	-2,304	Phosphoenolpyruvate-dihydroxyacetone phosphotransferase (EC 2.7.1.121), glycolysis	Carbohydrate metabolism
**EcN1917_2438**	-2,303	Inosine-uridine preferring nucleoside hydrolase (EC 3.2.2.1), metal binding in metabolic process	Metal/ion binding
**EcN1917_0298**	-2,300	putative membrane protein	Membrane protein
**EcN1917_0646**	-2,292	2-(5''-triphosphoribosyl)-3'-dephosphocoenzyme- A synthase (EC 2.7.8.25), prosthetic group biosynthetic process	Other metabolism and biosynthesis related proteins
**EcN1917_2096**	-2,271	Uncharacterised protein	Uncharachterised protein
**EcN1917_1786**	-2,266	putative oxidoreductase 2C Fe-S subunit, metal/ion binding	Metal/ion binding
**EcN1917_3528**	-2,260	Tagatose-6-phosphate kinase AgaZ (EC 2.7.1.144), galactitol metabolic process	Carbohydrate metabolism
**EcN1917_1839**	-2,255	Ribulosamine/erythrulosamine 3-kinase potentially involved in protein deglycation	Carbohydrate metabolism
**EcN1917_3144**	-2,246	Xanthine permease, involved in metabolic processes	Other metabolism and biosynthesis related proteins
**EcN1917_3524**	-2,228	D-galactarate dehydratase (EC 4.2.1.42), carbohydrate metabolism	Carbohydrate metabolism
**EcN1917_3126**	-2,227	Uncharacterised sigma-54-dependent transcriptional regulator YgeV, transcription	Transcription and translation
**EcN1917_3614**	-2,224	N-acetylneuraminate lyase (EC 4.1.3.3), carbohydrate metabolism	Carbohydrate metabolism
**EcN1917_1597**	-2,202	Transcriptional regulator 2C GntR family, transcription	Transcription and translation

**EcN1917_3048**	-2,201	L-fuculose phosphate aldolase (EC 4.1.2.17), carbohydrate metabolism	Carbohydrate metabolism
**EcN1917_3124**	-2,188	Xanthine dehydrogenase 2C FAD binding subunit (EC 1.17.1.4), nucleotide metabolism	Nucleotide, RNA, DNA metabolism
**EcN1917_3388**	-2,176	Predicted oxidoreductases (related to aryl-alcohol dehydrogenases), electron transport chain	Electron transport chain
**EcN1917_2073**	-2,166	Ferritin-like protein 2, metal binding	Metal/ion binding
**EcN1917_3612**	-2,158	N-acetylmannosamine-6-phosphate 2-epimerase (EC 5.1.3.9), carbohydrate metabolism	Carbohydrate metabolism
**EcN1917_2072**	-2,148	L-arabinose-binding periplasmic protein precursor AraF (TC 3.A.1.2.2), L-arabinose transport	Carbohydrate metabolism
**EcN1917_3935**	-2,137	Cytochrome c551 peroxidase (EC 1.11.1.5), electron transport chain	Electron transport chain
**EcN1917_1556**	-2,121	Aldo-keto reductase, electron transport chain	Electron transport chain
**EcN1917_3925**	-2,120	Chaperone HdeB, acid stress	Stress related protein
**EcN1917_3926**	-2,115	Chaperone HdeA, acid stress	Stress related protein
**EcN1917_3130**	-2,097	Dihydropyrimidinase (EC 3.5.2.2), metal/ion binding	Metal/ion binding
**EcN1917_1384**	-2,096	Uncharacterised protein	Uncharachterised protein
**EcN1917_0030**	-2,090	Uncharacterised protein	Uncharachterised protein
**EcN1917_1613**	-2,089	mobile element protein	Uncharachterised protein
**EcN1917_4609**	-2,087	Uncharacterised protein	Uncharachterised protein
**EcN1917_3830**	-2,078	Fimbrial protein	Fimbrial protein
**EcN1917_0442**	-2,067	Putative inner membrane protein	Membrane protein
**EcN1917_2824**	-2,059	Uncharacterised protein	Uncharachterised protein
**EcN1917_4889**	-2,039	Uncharacterised protein	Uncharachterised protein

**EcN1917_2425**	-2,038	Galactose/methyl galactoside ABC transport system 2C ATP-binding protein MglA (EC 3.6.3.17), galactose transport system	Carbohydrate metabolism
**EcN1917_0632**	-2,032	Hypothetical small protein yjiX /replicative DNA helicase, DNA metabolism	Nucleotide, RNA, DNA metabolism
**EcN1917_3522**	-2,029	D-glucarate permease, carbohydrate metabolism and transport related genes	Carbohydrate metabolism
**EcN1917_3534**	-2,017	Galactosamine-6-phosphate isomerase, carbohydrate metabolism	Carbohydrate metabolism

Table S3: Details of the genes upregulated in EcN when incubated with T4 phages after 2 h (log_2foldchange>2, padj<0.05)

**Upregulated genes**	**log_2foldchange**	**Functional prediction**	**Functional group**
**EcN1917_1772**	4,9758	Putative transport protein	Membrane protein or transport related genes
**EcN1917_3763**	3,4163	TsgA protein homolog, involved in transmembrane transport	Membrane protein or transport related genes
**EcN1917_4067**	2,7274	Putative inner membrane protein	Membrane protein or transport related genes
**EcN1917_2418**	2,6690	Cytidine deaminase, nucleoside metabolism	Nucleotide, RNA, DNA metabolism
**EcN1917_2679**	2,6261	Nucleoside permease NupC, nucleotide transport	Nucleotide, RNA, DNA metabolism
**EcN1917_3557**	2,5741	DEAD-box ATP-dependent RNA helicase CshA, ribosome biogenesis	Transcription or translation

**EcN1917_0131**	2,5133	PQQ-dependent glucose dehydrogenase (EC 1.1.5.2), pentose phosphate pathway	Carbohydrate metabolism
**EcN1917_4569**	2,4706	YjcB protein, membrane protein	Membrane protein or transport related genes
**EcN1917_1507**	2,4084	ABC-type sugar transport system periplasmic binding protein YcjN	Membrane protein or transport related genes
**EcN1917_2337**	2,4051	Polysaccharide export lipoprotein Wza, LPS synthesis	Membrane protein or transport related genes
**EcN1917_4973**	2,4040	Thymidine phosphorylase (EC 2.4.2.4), nucleoside metabolism	Nucleotide, RNA, DNA metabolism
**EcN1917_1631**	2,3324	Bdm protein, positive regulation of bacterial-type flagellum assembly	Flagella protein
**EcN1917_4972**	2,2810	Deoxyribose-phosphate aldolase (EC 4.1.2.4), involved in deoxyribose phosphate catabolic process	Nucleotide, RNA, DNA metabolism
**EcN1917_0626**	2,2317	Isochorismate synthase (EC 5.4.4.2), siderophore biosynthesis	Siderophore biosynthesis
**EcN1917_0474**	2,2233	Nucleoside-specific channel-forming protein Tsx precursor	Nucleotide, RNA, DNA metabolism
**EcN1917_3698**	2,1533	LSU ribosomal protein L15p (L27Ae)	Transcription or translation
**EcN1917_2278**	2,1440	Putrescine importer	Membrane protein or transport related genes
**EcN1917_3978**	2,1340	Outer membrane protein A precursor	Membrane protein or transport related genes
**EcN1917_4843**	2,1094	LysR family transcriptional regulator	Transcription or translation

**EcN1917_1516**	2,0761	Outer membrane protein G precursor	Membrane protein or transport related genes
**EcN1917_1275**	2,0746	Spermidine putrescine ABC transporter permease component PotB	Membrane protein or transport related genes
**EcN1917_3579**	2,0602	Permease of the drug/metabolite transporter (DMT) superfamily	Membrane protein or transport related genes
**EcN1917_0624**	2,0518	Enterobactin exporter EntS	Membrane protein or transport related genes
**EcN1917_0059**	2,0513	Ribosomal large subunit pseudo-uridine synthase A	Transcription or translation
**EcN1917_3693**	2,0468	SSU ribosomal protein S4p (S9e)	Transcription or translation
**EcN1917_3710**	2,0467	LSU ribosomal protein L16p (L10e)	Transcription or translation
**EcN1917_3657**	2,0434	DNA-binding protein Fis	Transcription or translation
**EcN1917_0936**	2,0347	SSU ribosomal protein S1p	Transcription or translation
**EcN1917_3709**	2,0342	LSU ribosomal protein L29p (L35e)	Transcription or translation
**EcN1917_3697**	2,0294	Preprotein translocase *secY* subunit, secretory pathway	Membrane protein or transport related genes
**EcN1917_1625**	2,0177	Formate dehydrogenase O beta subunit (EC 1.2.1.2), electron carrier activity	Transcription or translation
**EcN1917_3692**	2,0050	DNA-directed RNA polymerase alpha subunit (EC 2.7.7.6), DNA templated transcription	Transcription or translation
**EcN1917_0821**	2,0030	ATP-dependent RNA helicase RhlE, ribosome assembly	Transcription or translation

References:

1. Andrews, S. (2010). FastQC: a quality control tool for high throughput sequence data. .
2. Edgar, R., Domrachev, M., and Lash, A.E. (2002). Gene Expression Omnibus: NCBI gene expression and hybridization array data repository. *Nucleic Acids Res* 30**,** 207-210.
3. Forstner, K.U., Vogel, J., and Sharma, C.M. (2014). READemption-a tool for the computational analysis of deep-sequencing-based transcriptome data. *Bioinformatics* 30**,** 3421-3423.
4. Freese, N.H., Norris, D.C., and Loraine, A.E. (2016). Integrated genome browser: visual analytics platform for genomics. *Bioinformatics* 32**,** 2089-2095.
5. Hoffmann, S., Otto, C., Kurtz, S., Sharma, C.M., Khaitovich, P., Vogel, J., Stadler, P.F., and Hackermuller, J. (2009). Fast mapping of short sequences with mismatches, insertions and deletions using index structures. *PLoS Comput Biol* 5**,** e1000502.
6. Love, M.I., Huber, W., and Anders, S. (2014). Moderated estimation of fold change and dispersion for RNA-seq data with DESeq2. *Genome Biol* 15**,** 550.
7. Martin, M. (2011). Cutadapt removes adapter sequences from high-throughput sequencing reads. *EMBnet.Journal,* 17**,** 3.
