# Supplementary material for: K5 Capsule and Lipopolysaccharide Are Important in Resistance to T4 Phage Attack in Probiotic E. coli Strain Nissle 1917
Source: Front Microbiol. 2019 Nov 29;10:2783. doi: 10.3389/fmicb.2019.02783 (PMC6895014; doi:10.3389/fmicb.2019.02783)
Supplement: FIGURE S5 — Comparison of LPS among different E. coli strains used in the study: Structural comparison of LPS from EcN and E. coli K-12 strain (A). The dotted line in the K-12 core between GlcN and Hep indicates GlcN being present only in some E. coli K-12 strains (Orskov et al., 1977). The O antigen structures of different E. coli strains used in this study were listed out (B). The abbreviations used in this figure are GlcN: N-acetylglucosamine, Man: mannose, Glu: glucose, Gal: galactose, Hep: L-glycero-D-manno heptose, Kdo: 3-deoxy-D-manno-oct- 2ulosonic acid, P: phosphate, Gal: N-acetylgalactosamine, Rhap: rhamno pyranose, GlcN(Slac): 2-acetamido-4-O-[(S)-1-carboxy-ethyl]-2-deoxy-D-glucose. [file Image_5.PDF]

Figure S5. Comparison of LPS among different *E. coli* strains used in the study:

Structural comparison of LPS from EcN and *E. coli* K-12 strain (A). The dotted line in the K-12 core between GlcN and Hep indicates GlcN being present only in some *E. coli* K-12 strains (Orskov et al., 1977). The O antigen structures of different *E. coli* strains used in this study were listed out (B). The abbreviations used in this figure are GlcN: N-acetylglucosamine, Man: mannose, Glu: glucose, Gal: galactose, Hep: L-glycero-D-manno heptose, Kdo: 3-deoxy-D-manno-oct-2ulosonic acid, P: phosphate, Gal: N-acetylgalactosamine, Rhap: rhamnopyranose, GlcN(Slac): 2-acetamido-4-O-[(S)-1-carboxy-ethyl]-2-deoxy-D-glucose.

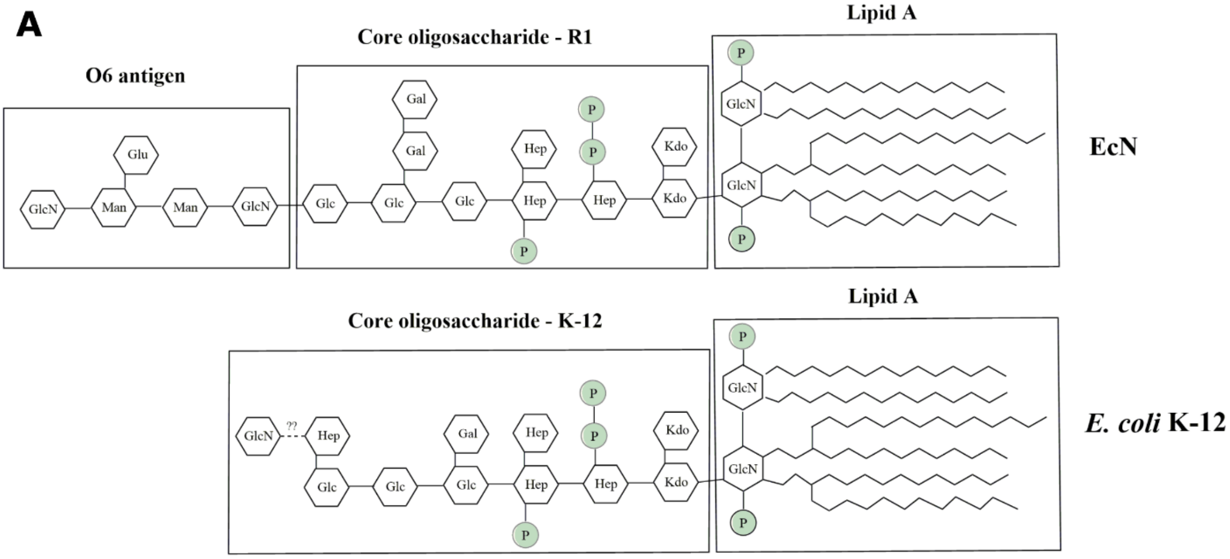

B

E. coli strains<br>(O ant:K ant:flagella type)	**O antigen structure**
**EcN (O6:K5:H1)**	Glu (on Man)<br>GlcN - Man - Man - GlcN –
**CFT073 (O6:K2:H1)**	Glu (on Man)<br>– GalN - Man - Man - GlcN –
**SE11 (O152:K-:H8)**	RhaP (on GlcN)<br>– GlcN - Glu - Glu - GlcN –
**SE15 (O150:K-:H5)**	Glu (on third Rhap)<br>– GlcN(Slac) - Rhap - Rhap - Rhap - GlcN –

LPS structures were adapted from (Grozdanov et al., 2002) for EcN; (Orskov et al., 1977;Washizaki et al., 2016) for *E. coli* K-12 MG1655; (Stenutz et al., 2006) for CFT073, SE11; (Perepelov et al., 2007) for SE15.

References:

Grozdanov, L., Zahringer, U., Blum-Oehler, G., Brade, L., Henne, A., Knirel, Y.A., Schombel, U., Schulze, J., Sonnenborn, U., Gottschalk, G., Hacker, J., Rietschel, E.T., and Dobrindt, U. (2002). A single nucleotide exchange in the wzy gene is responsible

for the semirough O6 lipopolysaccharide phenotype and serum sensitivity of Escherichia coli strain Nissle 1917. *J Bacteriol* 184**,** 5912-5925.

Orskov, I., Orskov, F., Jann, B., and Jann, K. (1977). Serology, chemistry, and genetics of O and K antigens of Escherichia coli. *Bacteriol Rev* 41**,** 667-710.

Perepelov, A.V., Han, W., Senchenkova, S.N., Shevelev, S.D., Shashkov, A.S., Feng, L., Liu, Y., Knirel, Y.A., and Wang, L. (2007). Structure of the O-polysaccharide of Escherichia coli O150 containing 2-acetamido-4-O-[(S)-1-carboxyethyl]-2-deoxy-d-glucose. *Carbohydr Res* 342**,** 648-652.

Stenutz, R., Weintraub, A., and Widmalm, G. (2006). The structures of Escherichia coli O-polysaccharide antigens. *FEMS Microbiol Rev* 30**,** 382-403.

Washizaki, A., Yonesaki, T., and Otsuka, Y. (2016). Characterization of the interactions between Escherichia coli receptors, LPS and OmpC, and bacteriophage T4 long tail fibers. *Microbiologyopen* 5**,** 1003-1015.
